# Circulating miRNA-21-5p as a diagnostic biomarker for pancreatic cancer: evidence from comprehensive miRNA expression profiling analysis and clinical validation

**DOI:** 10.1038/s41598-017-01904-z

**Published:** 2017-05-10

**Authors:** Kai Qu, Xing Zhang, Ting Lin, Tian Liu, Zhixin Wang, Sushun Liu, Lei Zhou, Jichao Wei, Hulin Chang, Ke Li, Zheng Wang, Chang Liu, Zheng Wu

**Affiliations:** 1 Department of Hepatobiliary Surgery, The First Affiliated Hospital of Xi’an Jiaotong University, Xi’an, 710061 China; 2 Department of Hematology, The Second Affiliated Hospital of Xi’an Jiaotong University, Xi’an, 710004 China; 3 Department of Hepatopancreatobiliary Surgery, Affiliated Hospital of Qinghai University, Xining, 810001 China; 4 Department of General Surgery, The Second Xiangya Hospital, Central South University, Changsha, 410011 China; 5 Department of Hepatobiliary Surgery, The Affiliated Hospital of Binzhou Medical University, Binzhou, 256603 China; 6 Department of Hepatobiliary Surgery, Qingdao Municipal Hospital, Qingdao, 266011 China; 7 Department of Hepatobiliary Surgery, Shaanxi Provincial People’s Hospital, Xi’an, 710068 China; 8 Department of Central Laboratory, Liaocheng People’s Hospital, Liaocheng, 252000 China

## Abstract

Pancreatic cancer (PC) is a highly fatal disease worldwide and is often misdiagnosed in its early stages. The exploration of novel non-invasive biomarkers will definitely benefit PC patients. Recently, circulating miRNAs in body fluids are emerging as non-invasive biomarkers for PC diagnosis. In this study, we first conducted comprehensive robust rank aggregation (RRA) analysis based on 21 published miRome profiling studies. We statistically identified and clinically validated a miRNA expression pattern in PC patients. These miRNAs consisted of four up-regulated (hsa-miR-21-5p, hsa-miR-31-5p, hsa-miR-210-3p and hsa-miR-155-5p) and three down-regulated miRNAs (hsa-miR-217, hsa-miR-148a-3p and hsa-miR-375). Among them, hsa-miR-21-5p was one of the most highly expressed miRNAs in the serum of PC patients. Our validation test further suggested a relatively high accuracy of serum hsa-miR-21-5p levels in the diagnosis of PC, with a sensitivity of 0.77 and a specificity of 0.80. Finally, a diagnostic meta-analysis based on 9 studies also revealed favorable sensitivity and specificity of circulating hsa-miR-21-5p for the diagnosis of PC (pooled sensitivity and specificity were 0.76 and 0.74, respectively), which was consistent with our findings. Taken together, as one of the most aberrantly expressed miRNAs in PC, circulating hsa-miR-21-5p might be a promising serum biomarker in patients with PC.

## Introduction

Pancreatic cancer (PC), which is one of the most common cancers worldwide, is a highly fatal disease with more than 200,000 people diagnosed every year^1^. The median overall survival of patients with PC is less than 1 year, and a 5-year overall survival rate is no more than 5%^2, 3^. Despite the implementation of curative treatments, which have to some extent, improved the poor prognosis of multiple tumor types over the years, the overwhelmingly high mortality of PC is far from satisfactory. Due to a lack of specific clinical symptoms, PC is rarely detected at an early stage and is usually fatal within months of diagnosis. Therefore, the exploration of novel biomarkers for early diagnosis, prognostic prediction and effective therapies will definitely benefit patients with PC.

MicroRNAs (miRNAs) are endogenous RNAs that are approximately 22 nt in length that regulate the expression and function of protein-coding RNAs^4^. Since overwhelming evidence has demonstrated that miRNAs facilitate tumor growth, invasion, angiogenesis, and immune evasion via the targeting of mRNAs, they have thus been demonstrated to be key molecular components of the cell^5^. As a family of molecules that are stable in tissue and body fluid samples, miRNAs were found to be differentially expressed during pancreatic carcinogenesis^6^. Therefore, miRNAs are considered promising biomarkers for the diagnosis of PC.

In the past decade, researchers have put forth great effort to identify tumor-specific miRNAs by high-throughput miRNA profiling techniques, including second-generation sequencing and microarray-based methods^7, 8^. The applications of high-throughput miRNA profiling have enabled researchers to identify a group of differentially expressed miRNAs in cancers, which have potential as biomarkers for diagnostic, prognostic and therapeutic applications^9^. However, inconsistent conclusions have been garnered among those miRNA profiling studies. The possible causes may include, but are not limited to, small specimen size, use of different technological platforms, and the application of different statistical methods and cut-off criteria for aberrantly expressed miRNAs. In order to overcome the limitations discussed above in the identification of tumor-specific miRNAs, a meta-analysis that applied the robust rank aggregation (RRA) method was specifically designed to compare several ranked gene lists and to identify the most common overlapping genes^10^. During the past several years, using the RRA method, a number of studies have attempted to investigate the miRNA expression patterns in cancers of the breast, lung, liver, kidney, bladder, colon, ovary, and nasopharyngeal tract^11–19^. In 2013, Ma *et al*. also successfully identified 10 aberrantly expressed miRNAs in PC, which might be potential biomarkers for cancer diagnosis and prognosis^20^.

The most ambitious aim of the above-mentioned studies was to develop tissue-based biomarkers for the early diagnosis of cancer. However, not all PC patients have operable disease, and many of them do not have available cancer tissues that can be used for miRNA detection. Recently, one hypothesis has clearly demonstrated that cancer cells contribute to the pool of circulating miRNAs, which allows for the detection of cancer-specific miRNAs in a patient’s circulation. Circulating miRNAs represent a rich resource of potential biomarkers for PC diagnosis^21, 22^. In the present study, we used a two-phase design to investigate the diagnostic accuracies of circulating miRNAs in patients with PC. In the first phase, we explored miRNA expression patterns in PC based on RRA methods and validated the most consistently aberrantly expressed miRNAs in our own cohort. In the second phase, we explored the expression levels of the above-listed miRNAs in serum samples and performed a meta-analysis to evaluate the diagnostic performance of circulating miRNAs in the diagnosis of PC.

## Materials and Methods

### Literature search strategy

To identify miRome profiling studies in PC, we conducted a two-step search strategy as followings: (1) we performed a literature search in the Web of Knowledge (http://login.webofknowledge.com), Scopus (www.scopus.com), and PubMed (http://www.ncbi.nlm.nih.gov/pubmed/) databases using search term TITLE-ABS-KEY ((mirna* OR microrna* OR mir-*) and profil* and pancrea* and (cancer* OR tumor* OR tumour*)) from article titles, abstracts, and keywords; (2) we carried out searches of microarray datasets in Gene Expression Omnibus (GEO, www.ncbi.nlm.nih.gov/geo/) and ArrayExpress (www.ebi.ac.uk/arrayexpress) when miRNA lists were not available in the publications. Besides, we also screened all citations of relevant studies to guarantee the relevant studies were not missed. Authors were also contacted when key data were not available.

### Inclusion and exclusion criteria

Title, abstracts of each studies had been viewed and full text of relevant studies had been carefully evaluated. Two investigators (Kai Qu and Xing Zhang) independently scanned above information of each study to avoid record error. The included studies should: (1) be original experimental studies comparing the miRNA expression between PC cancer tissue and adjacent noncancerous tissue in human; (2) employ at least one miRome profiling technique such as high-throughput (96- or 384-well microplates based) quantitative polymerase chain reaction (qPCR), microarray or next-generation sequencing (NGS) methods; (3) include available up- and/or down-regulated miRNA lists according to respective cut-off criteria; (4) be published in English language. Meanwhile, exclusion criteria include: (1) studies that conducted only on cell lines or animal models; (2) studies measured only a few preselected individual miRNAs or a set of preselected miRNAs; (3) up- and/or down-regulated miRNA lists were still not available after screening microarray datasets and contacting with authors; (4) studies in non-English languages.

### Robust rank aggregation analysis

All miRNA names extracted from each study were standardized according to miRBase release 21 (http://www.mirbase.org/). Non-human miRNA (including viral, mouse and rat miRNAs), mRNA and lncRNA probes were not included. The ranked lists of normalized up- and down-regulated miRNAs were separately recorded and used for the following RRA analysis. RRA analysis was performed with an R package “Robust Rank Aggregation” (freely available in the comprehensive R Archive Network website, http://cran.r-project.org/) to identify meta-signature miRNAs in PC. The RRA method compared each actual piece of data with a null model expecting random ordering. To evaluate how much more highly it was ranked than expected, a *P*-value was assigned to each miRNA. A Bonferroni correction was performed to reduce false-positive results.

### Prediction of function of miRNAs

To predict the biological function of a particular miRNA, Kyoto Encyclopedia of Genes and Genomes (KEGG) pathway enrichment analysis was performed using the DAVID (https://david.ncifcrf.gov/) and GeneCodis tools (http://genecodis.dacya.ucm.es/). The false discovery rate (FDR)-corrected *P*-values of each miRNA obtained by KEGG pathway enrichment analysis were visualized as a heat map. Pearson correlation and average linkage were used to cluster the enriched KEGG pathways. In addition, the miRPath algorithm was employed by using DIANA tools (freely available in http://www.microrna.gr/miRPathv2) to predict the combinatorial effects of multiple miRNAs in KEGG pathways.

### Selection and characteristics of miRNA expression datasets

To validate the expression levels of meta-signature miRNAs in PC tissues, we searched miRNA expression datasets in the Gene Expression Omnibus database (GEO, www.ncbi.nlm.nih.gov/geo/) using the search terms (pancrea*) AND (cancer*) AND (mirna* OR microrna* OR mir-*). In all, 25 non-coding RNA profile series were found in the GEO database. The dataset GSE24279 had the largest number of tissue samples, which included 136 PC samples, 27 pancreatitis samples and 22 normal controls. In this dataset, each miRNA was measured in seven replicates and the median value was computed. Moreover, the dataset GSE59856, which included 100 serum samples obtained from PC patients and 21 serum samples obtained from patients without malignancies, was also selected for the exploration of the diagnostic values of meta-signature miRNAs.

### Diagnostic meta-analysis

A diagnostic meta-analysis was then conducted using STATA12.0 software to validate the diagnostic value of circulating miR-21 in body fluid samples from patients with PC. A literature search was performed using the Web of Knowledge, Scopus and Pubmed databases. The search terms were as follows: (microrna-21 OR mirna-21 OR mir-21) and (pancrea* and (cancer* OR tumor* OR tumour* OR carcinoma)) and (sensitivity OR specificity). After a literature screen, data were then extracted from each eligible study, including basic characteristics and diagnostic results. To assess the diagnostic effects, pooled sensitivity, specificity and AUC were calculated based on bivariate meta-analysis models. The summary receiver operating characteristic (ROC) curve was then computed to depict the pooled sensitivity and specificity of each study. Deek’s funnel plot was used for the evaluation of publication bias. A *P*-value less than 0.10 indicated significant publication bias.

### Patient recruitment and sample collection

In all, 95 PC patients were recruited from six medical centers in China, including the Affiliated Hospital of Qinghai University, the Second Xiangya Hospital of Central South University, the Affiliated Hospital of Binzhou Medical University, Qingdao Municipal Hospital, Shaanxi Provincial People’s Hospital, and Liaocheng People’s Hospital. All included individuals belonged to the Chinese Han ethnic group. Paired cancer and noncancerous adjacent tissue specimens were collected intra-operatively and were stored at −80 °C until further use. In addition, we also collected serum samples from 56 of those recruited patients and from 15 healthy volunteers. After collection, at least 2 mL of serum was immediately cleared of debris by brief centrifugation and was then stored at −80 °C. This study was approved by the Institutional Review Board for Human Research of the First Affiliated Hospital of Xi’an Jiaotong University. All recruited participants provided informed consent. The clinical-pathological characteristics of all participants recruited for the study are summarized in Table S1.

### Total RNA extraction and quantitative real-time PCR

As previously reported^23^, total RNA derived from tissue samples was extracted using TRIzol Reagent (Invitrogen, Carlsbad, CA, USA), while total RNA derived from serum samples was isolated using TRIzol LS Reagent (Invitrogen, Carlsbad, CA, USA. The expression level of each selective miRNA was determined by a SYBR® PrimeScript^TM^ miRNA RT-PCR Kit and a SYBR® Premix Ex Taq^TM^ Kit (TaKaRa Biotechnology, Dalian, China). The miRNA expression was assayed in triplicate and was normalized to corresponding housekeeping miRNAs, RNU6 (for tissue miRNAs) or cel-miR-39 (for serum miRNAs).

### Statistical analysis

Statistical analyses were performed using SPSS 11.0 software (SPSS Inc, Chicago, IL, USA). All experiments in this study were repeated three times. The data were presented as mean ± standard error of measurement (SEM) and were performed using a Student’s *t*-test. *P* < 0.05 was considered statistically significant.

## Results

### Literature selection and characteristics of eligible studies

**Figure 1:**
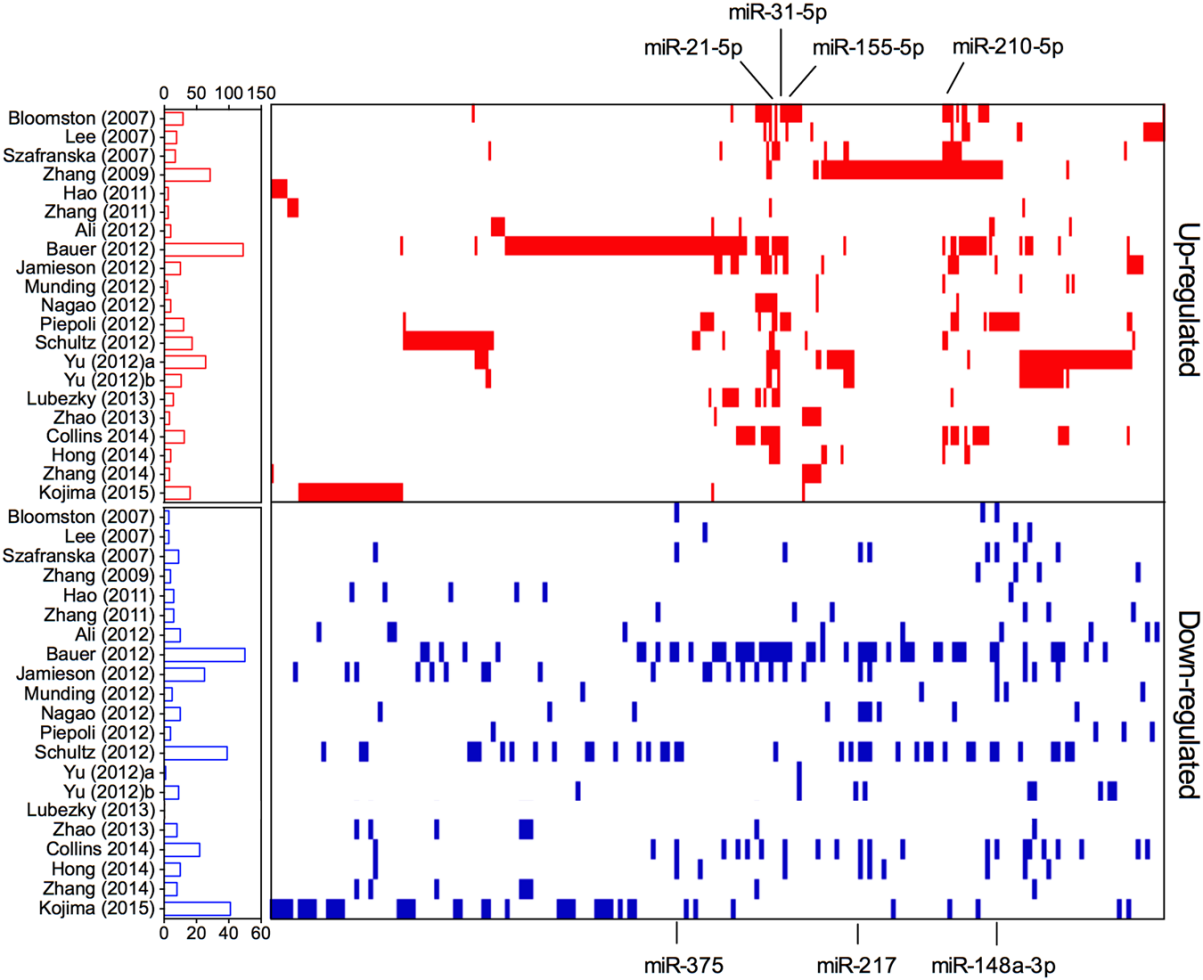
Distribution of miRNA alterations in PC as reported in 21 miRome profiling studies. Short red indicated up-regulated miRNAs and blue vertical bars indicated down-regulated miRNAs. The number of miRNAs in each study is graphically depicted on the left. The positions of meta-signature miRNAs have been marked.

We conducted a literature search according to the search criteria, and a flow diagram that shows a schematic of the process of article selection with specific reasons for those selections is shown in Figure S1. Briefly, 701 potentially relevant articles were obtained after the initial search, and 494 of them were excluded after the application of further inclusion/exclusion criteria. Finally, 21 pancreatic cancer miRNA expression profiling studies from 20 published articles were included^24–43^. All of the articles were published between the years 2007 and 2015. The basic characteristics of all 21 included studies, including the first author, date of publication, study period, country of origin and ethnicity of the recruited patients, sample type, detection platforms, total number of detected miRNAs and cut-off criteria, are summarized in Table S2. Across the studies, the number of samples investigated ranged from 9 to 211, and our pooled dataset finally included a total of 905 cancerous and 449 noncancerous samples. The majority (16/21, 76.2%) of the miRnome profiling studies used the microarray method, and the number of miRNA probes ranged from 281 to 3100. The number of aberrantly expressed miRNAs varied among individual studies (range, 3 to 122 miRNAs) and the lists of highly frequent aberrantly expressed miRNAs from individual miRNA profiling studies were substantially different (Fig. 1).

### Identification of meta-signature miRNAs in PC

**Figure 2:**
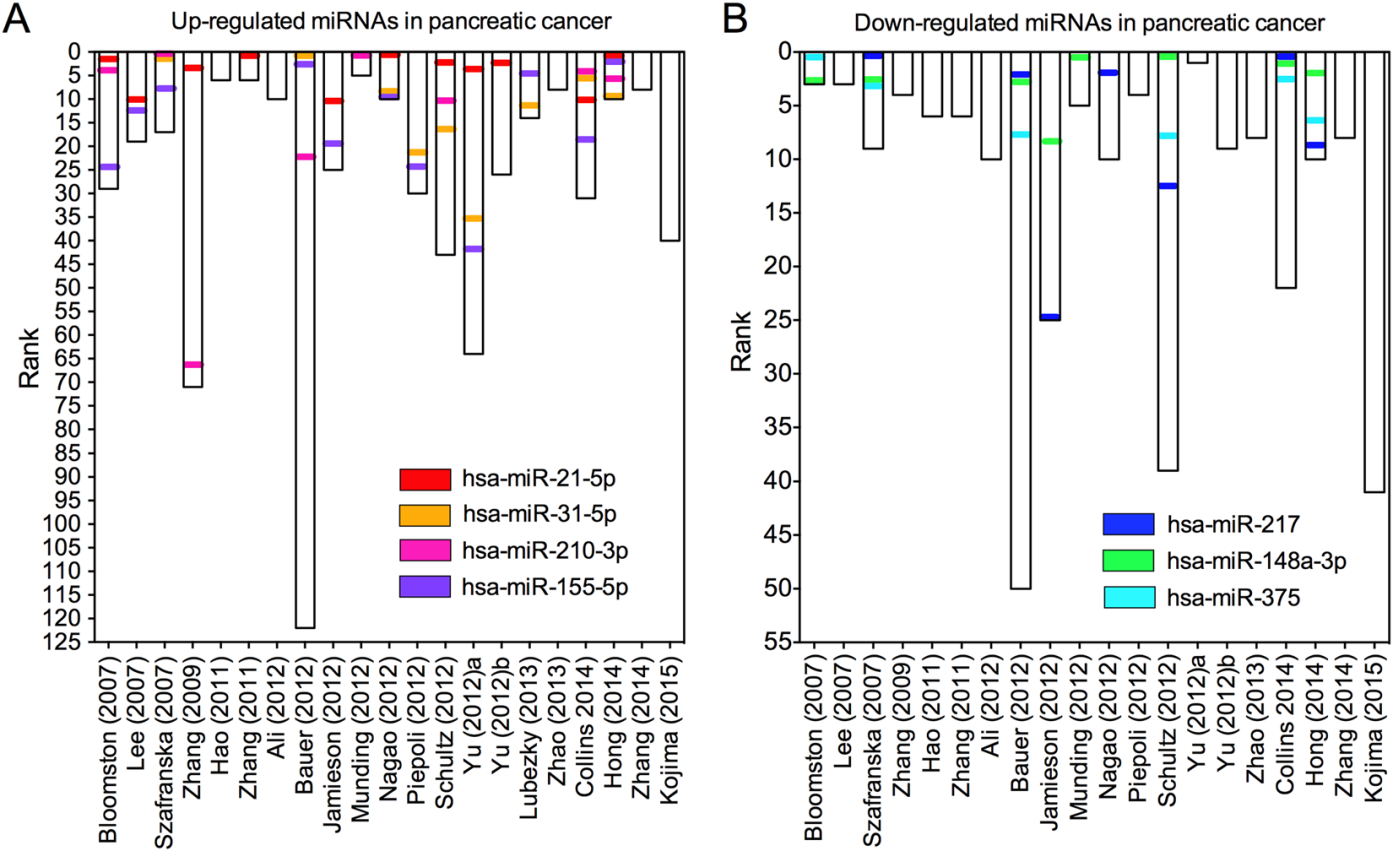
Ranks for meta-signature miRNAs in 21 miRome profiling studies. The ranks for four up-regulated miRNAs (hsa-miR-21-5p, hsa-miR-31-5p, hsa-miR-210-3p and hsa-miR-155-5p) (**A**) and three down-regulated miRNAs (hsa-miR-217, hsa-miR-148a-3p and hsa-miR-375) (**B**) in each of the enrolled study were depicted. Each column represents one of the 21 miRome profiling studies. The rank of miRNAs in each study is graphically depicted by different colors.

In all, 499 aberrantly expressed miRNAs were recorded, including 388 up-regulated and 189 down-regulated miRNAs. It should be mentioned that discordant alteration of 78 miRNAs was seen in lists of both up- and down-regulated miRNAs, which indicates the presence of inter-laboratory variation among different miRNA profiling studies. In Fig. 1, we listed all aberrantly expressed miRNAs; the red vertical bars indicate up-regulated miRNAs and the blue vertical bars indicate down-regulated miRNAs. Among them, 100 up-regulated (100/388, 25.8%) and 15 down-regulated (15/189, 7.9%) miRNAs were recorded in more than three studies; these were then categorized as highly frequent aberrantly expressed miRNAs. When these miRNAs were analyzed, we found no overlapping miRNAs between the lists of up- and down-regulated miRNAs, which suggests that a comprehensive analysis based on all relevant studies might provide more reliable evidence. We therefore performed a meta-analysis using the robust rank aggregation method according to previously published guidelines^10^. We identified a statistically significant meta-signature of four up-regulated miRNAs (hsa-miR-21-5p, hsa-miR-31-5p, hsa-miR-210-3p and hsa-miR-155-5p) and three down-regulated miRNAs (hsa-miR-217, hsa-miR-148a-3p and hsa-miR-375) in PC, with *P*-values that ranged from 1.09E-05 to 7.41E-10 and Bonferroni-corrected *P*-values that ranged from 3.39E-02 to 2.30E-06 (Table 1). All meta-signature miRNAs were reported more than 6 times with relatively high-rank scores in each study (Fig. 2), which provided reliable evidence for the following analysis.

**Table 1:** Meta-signature miRNAs in PC tissue.

microRNA	Chromosome	*P* value	Corrected *P* value	Studies
Up-regulated				
hsa-miR-21-5p	17q23.1	7.41E-10	2.30E-06	11
hsa-miR-31-5p	9p21.3	4.61E-07	1.43E-03	9
hsa-miR-210-3p	11p15.5	1.27E-06	3.94E-03	8
hsa-miR-155-5p	21q21.3	1.09E-05	3.39E-02	11
Down-regulated				
hsa-miR-217	2p16.1	1.69E-07	5.25E-04	7
hsa-miR-148a-3p	7p15.2	1.01E-06	3.13E-03	8
hsa-miR-375	2q35	5.43E-06	1.68E-02	6

### Prediction of function of meta-signature miRNAs

**Figure 3:**
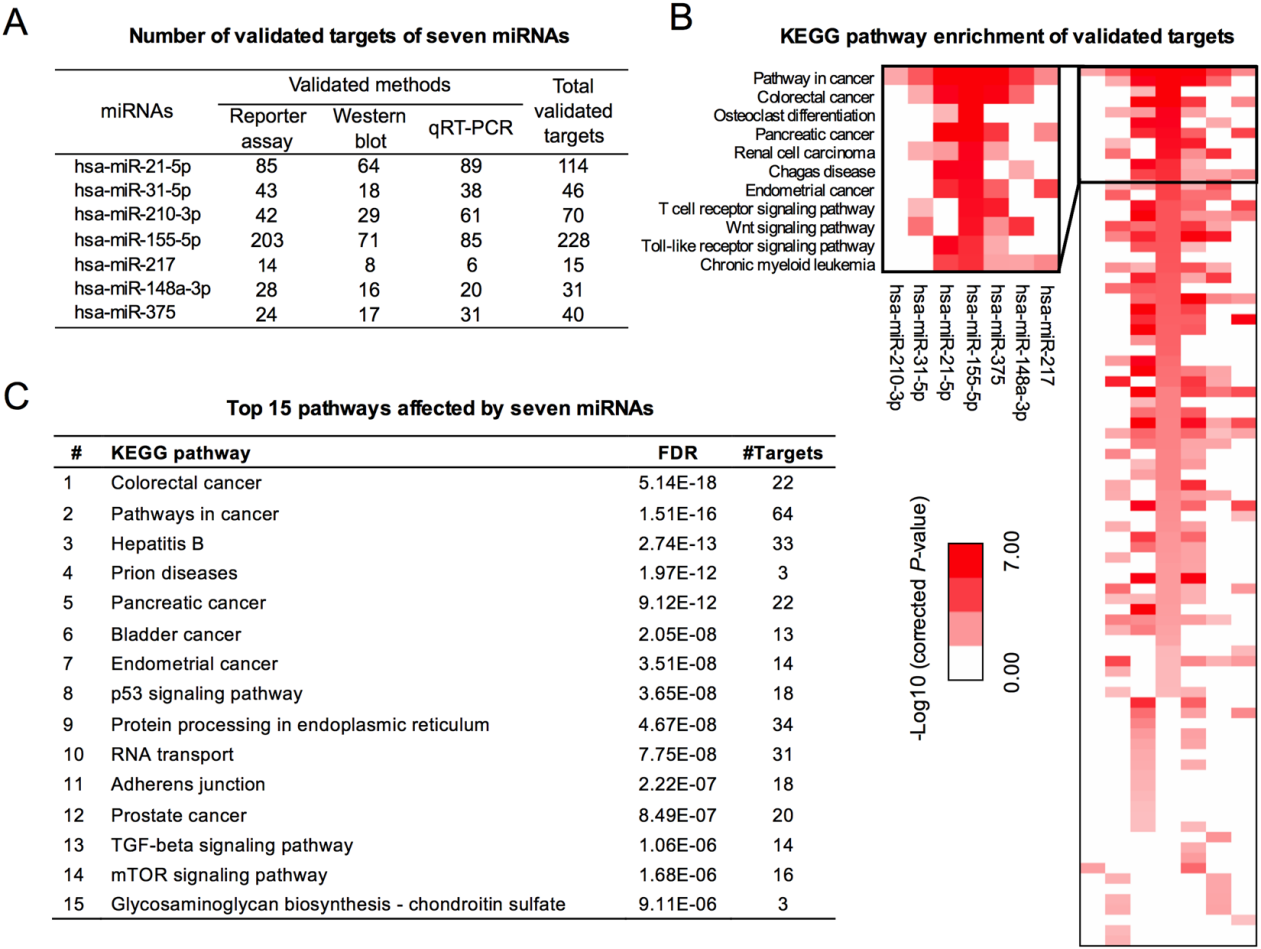
Target prediction and pathway enrichment analysis of seven meta-signature miRNAs. (**A**) Validated targets of seven meta-signature miRNAs. (**B**) Heatmap of the pathway enrichment of validated seven meta-signature miRNAs validated targets. Rows: pathways; Columns: miRNAs. Range of colors (deep red to white) shows the range of expression values (high to low). (**C**) The top 15 saturated pathways affected by seven meta-signature miRNAs.

It has been widely accepted that the primary biological function of miRNAs is to regulate gene expression by base-pairing to specific targets. Therefore, to predict the biological functions of meta-signature miRNAs identified by the RRA method, we performed pathway enrichment analysis of miRNA-specific targets. To avoid the inclusion of non-specific or even erroneous targets, we only selected targets with “strong evidence”, which means that those targets were experimentally validated by at least one of the following methods: reporter assay, western blot or qRT-PCR. As listed in Fig. 3A, the validated target number of each miRNA ranged from 15 to 228. The targets of each miRNA are presented in greater detail in Table S3. KEGG pathway enrichment analysis revealed that most miRNAs were closely related to cancer-related pathways, including pancreatic cancer-related pathways (Fig. 3B). Moreover, an analysis of the combinatorial effects of seven meta-signature miRNAs also confirmed the predictions described above. A rank list of KEGG pathways that were affected in a combinatorial manner by seven miRNAs was presented, and a pancreatic cancer-related pathway ranked fifth with an FDR of 9.12E-12 (Fig. 3C and Table S4). Moreover, a Kaplan-Meier survival analysis based on SurvMicro (http://bioinformatica.mty.itesm.mx/SurvMicro) also suggested that aberrant expression of those seven miRNAs significantly affected the overall survival of PC patients (Figure S2), which provides indirect evidence of the association between the seven meta-signature miRNAs and PC.

### Validation of meta-signature miRNAs in PC tissue samples

**Figure 4:**
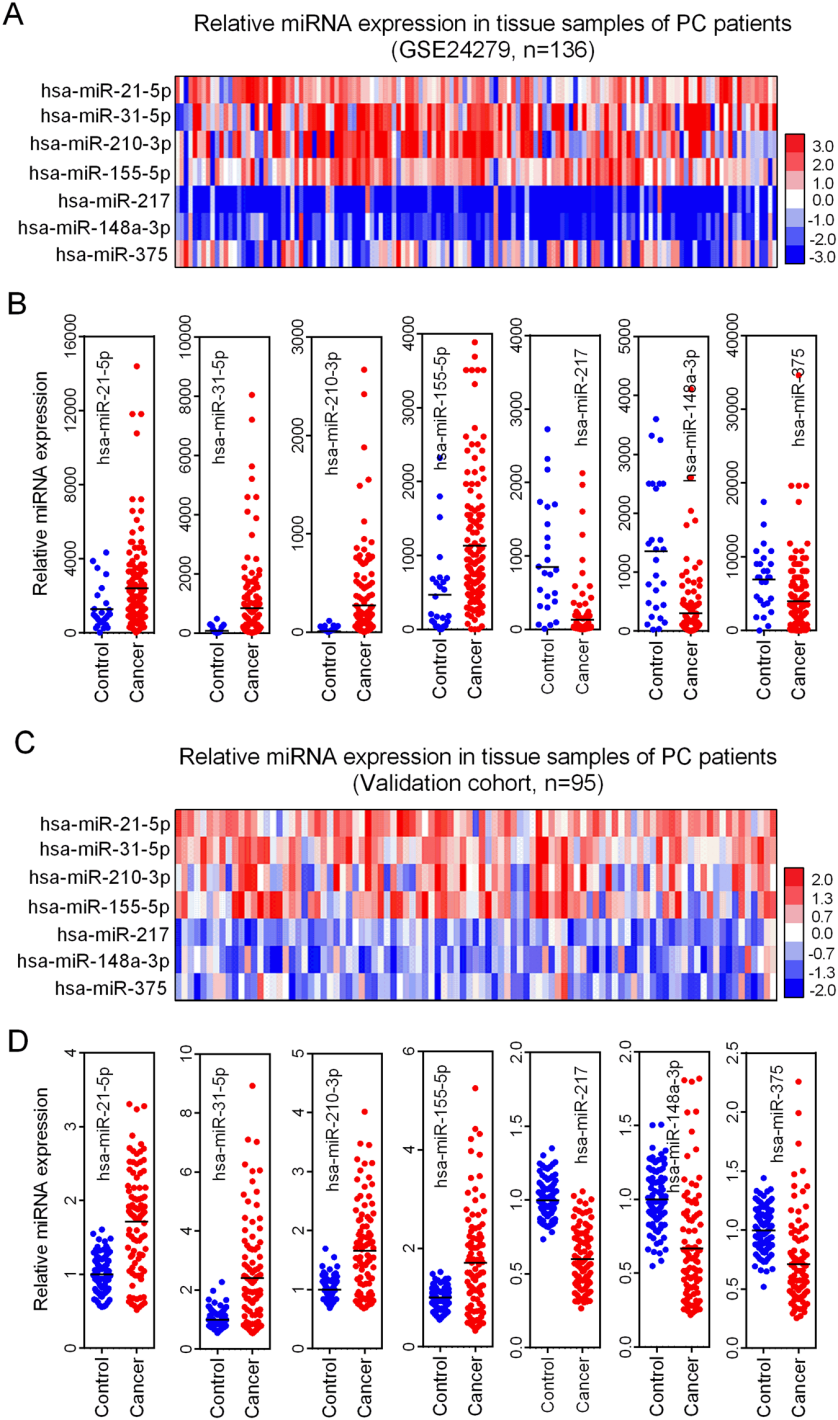
Meta-signature miRNAs were aberrantly expressed in PC tissue samples. (**A**) The expression of seven meta-signature miRNA in public datasets (GSE24279) was presented as heatmap. (**B**) The expression alteration of each meta-signature miRNA between PC and non-cancer groups in GSE24279. (**C**) The expression of seven meta-signature miRNA in our validation cohort was presented as heatmap. (**D**) The expression alteration of each meta-signature miRNA between PC and non-cancer groups in our validation cohort. PC: pancreatic cancer.

We used both public datasets and our own to further explore the expression pattern of seven meta-signature miRNAs in PC patients. The public dataset (GSE24279), which contains 136 PC tissue samples, was downloaded from the GEO database. Tissue expression of hsa-miR-21-5p, hsa-miR-31-5p, hsa-miR-210-3p and hsa-miR-155-5p was shown to be up-regulated in most patients with PC, whereas the tissue expression of hsa-miR-217, hsa-miR-148a-3p and hsa-miR-375 was shown to be down-regulated (Fig. 4A). The comparisons of the expression levels of seven meta-signature miRNAs between the cancer and control groups revealed that the differences were statistically significant (all *P* < 0.05, Fig. 4B). In our validation cohort, the expression pattern of seven miRNAs was consistent with that in the GSE24279 dataset (Fig. 4C,D). Our validation results further supplied evidence that those seven miRNAs might be potential biomarkers that may be used for the diagnosis of PC.

### The expression levels of miRNAs in PC serum samples

**Figure 5:**
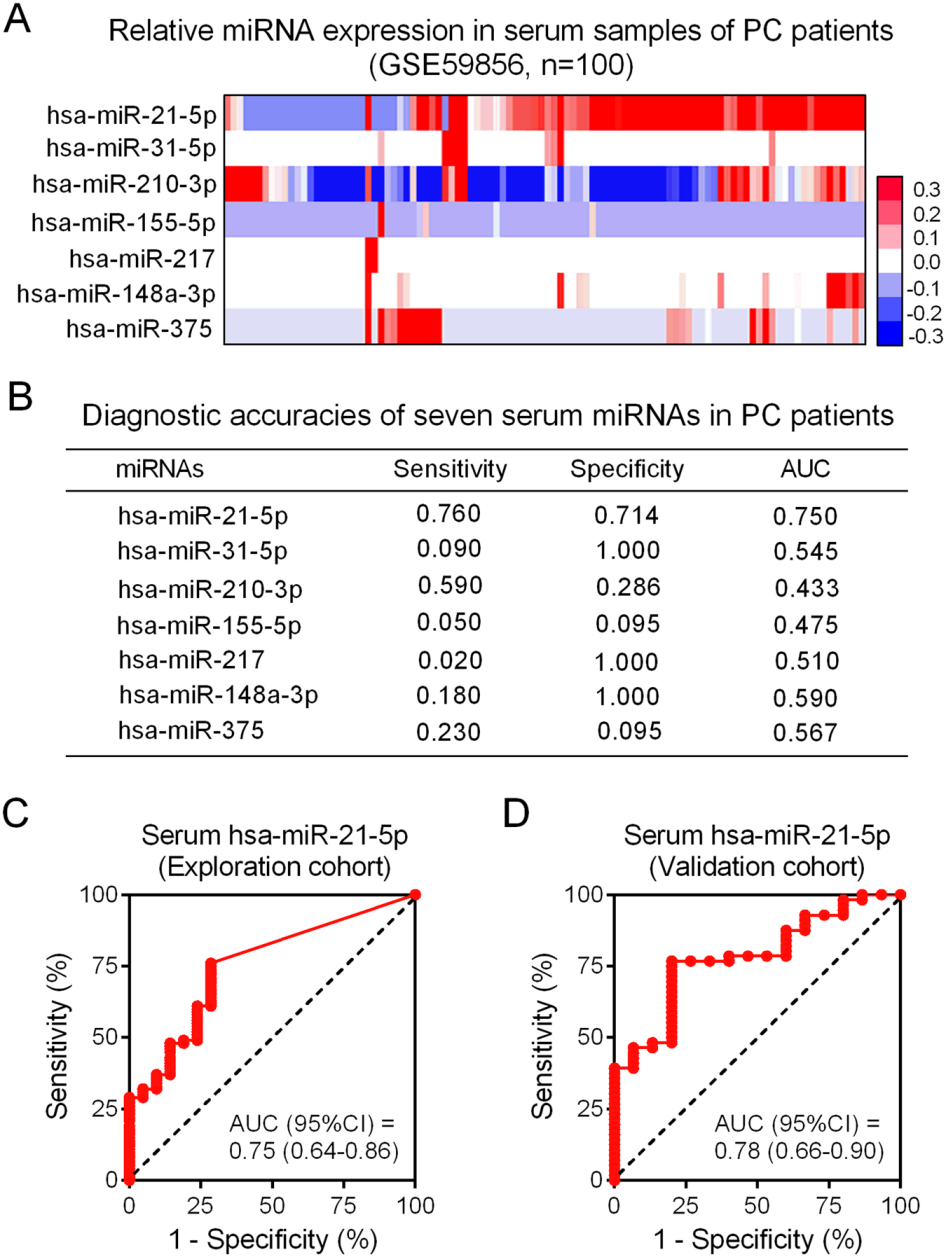
The expression and diagnostic accuracy of hsa-miR-21-5p in serum samples. (**A**) The expression alteration of seven meta-signature miRNAs in serum samples (GSE59856). (**B**) Diagnostic test of each meta-signature miRNA by using dataset GSE59856. (**C**) and (**D**) Receiver operating characteristic (ROC) curve was conducted to evaluate the diagnostic accuracy of serum hsa-miR-21-5p in GSE59856 dataset and our validation group. PC: pancreatic cancer; Sen, sensitivity; Spec, specificity; AUC, area under ROC curve.

It has been clearly demonstrated by our group and by others that miRNAs^23^, as short and stable non-coding RNAs, might be specifically secreted into body fluids by cancer cells and may, therefore, be potential biomarkers for cancer diagnosis. Based on the above observation, we hypothesized that these seven miRNAs are also aberrantly expressed in serum samples, which are much easier to collect and in which miRNAs are easier to detect compared with tissue samples. We conducted serum miRNA expression profiling using the GSE59856 dataset and found that only hsa-miR-21-5p could be detected in the majority of serum samples (Fig. 5A). We then calculated the diagnostic values of each miRNA, including the sensitivity, specificity and the area under the receiver operating characteristic (ROC) curve (AUC) (Fig. 5B). Circulating hsa-miR-21-5p had the best diagnostic accuracy among the seven miRNAs (Fig. 5B,C). Next, we evaluated the diagnostic performance of circulating hsa-miR-21-5p in our validation cohort (containing a total of 71 serum samples from 56 PC patients and 15 healthy volunteers). We found that the expression level of hsa-miR-21-5p in the serum was significantly correlated with the level in the corresponding tissue (Figure S3). Circulating hsa-miR-21-5p had a relatively high diagnostic accuracy in the diagnosis of PC, with a sensitivity of 0.77, a specificity of 0.80 and an AUC of 0.78 (95% CI: 0.66–0.90) (Fig. 5D).

### Circulating hsa-miR-21-5p is a potential diagnostic biomarker of PC

**Figure 6:**
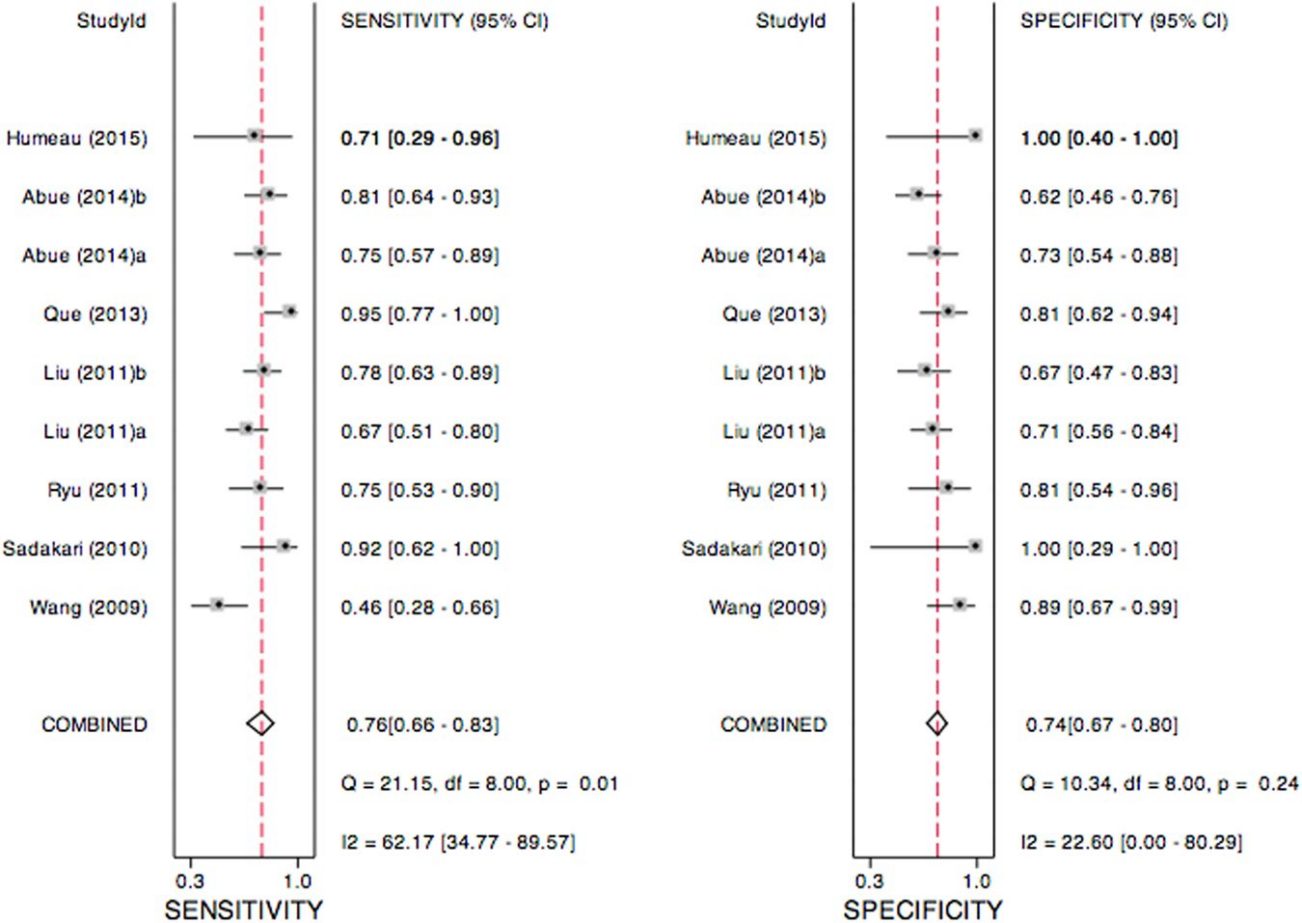
Forest plots showing the sensitivity and specificity of circulating hsa-miR-21-5p in the diagnosis of PC. (**A**) Forest plot showing the sensitivity of circulating hsa-miR-21-5p in the diagnosis of PC. (**B**) Forest plot showing the specificity of circulating hsa-miR-21-5p in the diagnosis of PC. 95% CI: 95% of confidence interval; PC: pancreatic cancer.

**Figure 7:**
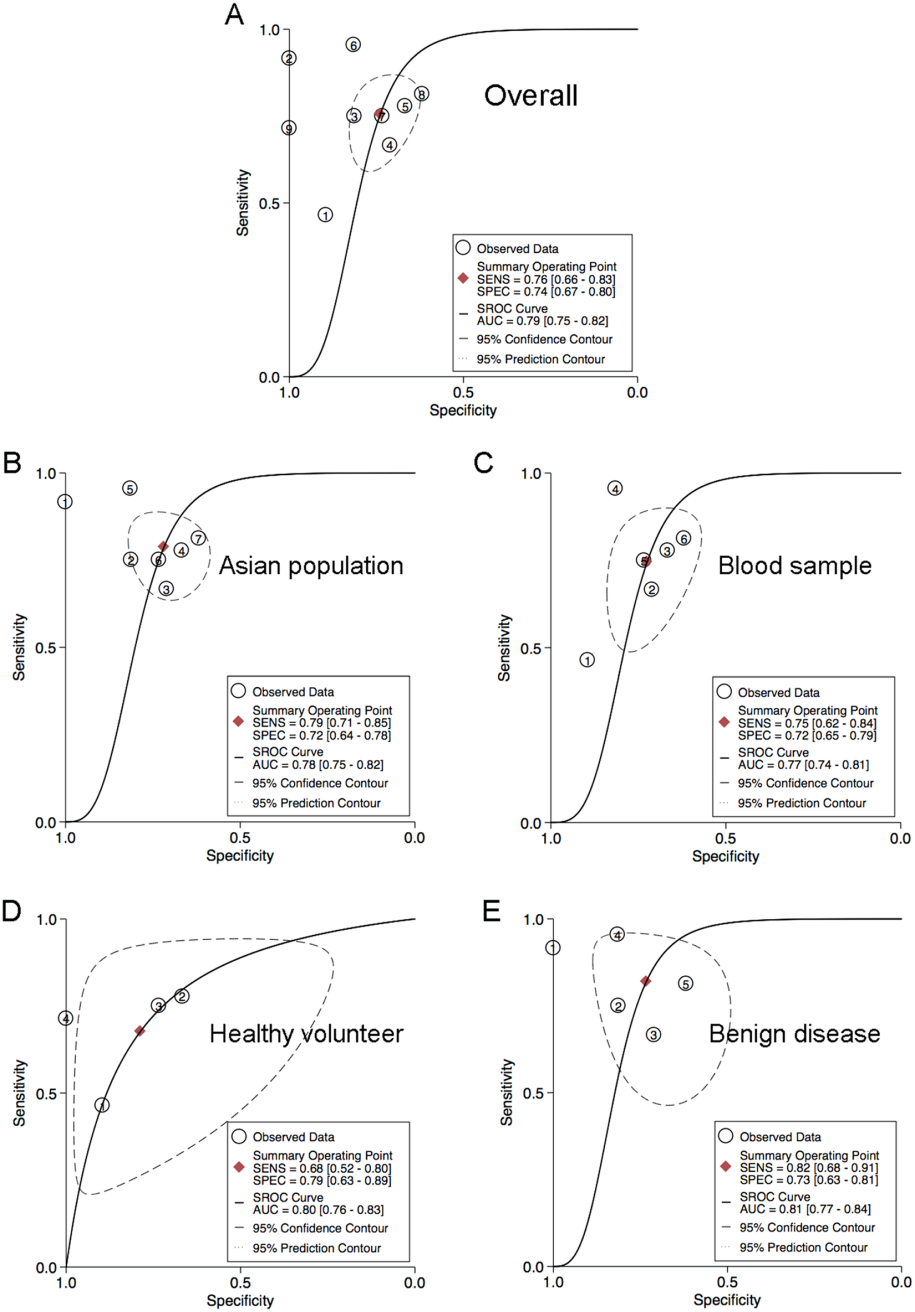
Summary receiver operating characteristic (SROC) curve of circulating hsa-miR-21-5p in PC diagnosis. (**A**) Summary receiver operating characteristic (SROC) curve of circulating hsa-miR-21-5p in the diagnosis of PC in overall patients. The summary receiver operating characteristic (SROC) curves of circulating hsa-miR-21-3p were conducted in subgroups, including Asian population (**B**), blood sample (**C**), healthy volunteer (**D**) or patients with benign disease (**E**) as controls. AUC: area under ROC curve; Sen: sensitivity; Spec: specificity; PC: pancreatic cancer.

To further comprehensively evaluate the diagnostic accuracy of circulating hsa-miR-21-5p in PC, we conducted a diagnostic meta-analysis based on 9 clinical studies derived from 7 articles^44–50^. A flow diagram that shows a schematic of the literature search process is presented in Figure S4. A total of 247 PC patients and 216 controls were included and the basic characteristics of each study are summarized in Table S5. It was revealed that the random-effects bivariate model was robust for this meta-analysis after goodness of fit and bivariate normality analysis were conducted (Figure S5). Additionally, Deek’s funnel plot asymmetry test suggested no publication bias in the meta-analysis (*P* = 0.15, Figure S6). The results of the diagnostic meta-analysis of circulating hsa-miR-21-5p are shown in Table 2. The pooled sensitivity, specificity and AUC of circulating hsa-miR-21-5p in the diagnosis of PC were 0.76, 0.74 and 0.79, respectively, which were all similar to the corresponding values in our study (Figs 6 and 7A). Moreover, we conducted subgroup analyses according to different clinical characteristics. We found various sensitivities and specificities across the different subgroups, with ranges of 0.68 to 0.82 and 0.72 to 0.79, respectively (Figure S7–S10). However, the summary AUC was found to be hardly influenced by clinical characteristics, with a range of 0.77 to 0.81 (Figs 6E and 7B), which suggests that circulating hsa-miR-21-5p might be a stable and relatively high-accuracy diagnostic biomarker for PC patients.

**Table 2:** Summary estimates of diagnostic test for circulating hsa-miR-21-5p in PC patients.

Analysis	No. of studies	SEN (95% CI)	SPEC (95% CI)	PLR (95% CI)	NLR (95% CI)	DOR (95% CI)	AUC (95% CI)
Overall	9	0.76 (0.66–0.83)	0.74 (0.67–0.80)	2.9 (2.3–3.7)	0.33 (0.23–0.47)	9 (5–14)	0.79 (0.75–0.82)
Outliers excluded	8	0.78 (0.71–0.85)	0.72 (0.65–0.79)	2.8 (2.2–3.7)	0.30 (0.21–0.42)	10 (5–17)	0.79 (0.76–0.83)
Ethnicity							
Asian	7	0.79 (0.71–0.85)	0.72 (0.64–0.78)	2.8 (2.1–3.7)	0.30 (0.20–0.43)	9 (5–17)	0.78 (0.75–0.82)
Sample sources							
Blood sample	6	0.75 (0.62–0.84)	0.72 (0.65–0.79)	2.7 (2.1–3.5)	0.35 (0.23–0.53)	8 (4–14)	0.77 (0.74–0.81)
Control sources							
Healthy volunteer	4	0.68 (0.52–0.80)	0.79 (0.63–0.89)	3.2 (1.8–5.5)	0.41 (0.28–0.60)	8 (4–16)	0.80 (0.76–0.83)
Benign disease	5	0.82 (0.68–0.91)	0.73 (0.63–0.81)	3.1 (2.1–4.6)	0.25 (0.13–0.47)	12 (5–33)	0.81 (0.77–0.84)

SEN, sensitivity; SPEC, specificity; PLR, positive likelihood ratio; NLR, positive likelihood ratio; DOR, diagnostic odds ratio; AUC, area under the ROC curve.

## Discussion

In the present study, using the RRA method, we first identified a miRNA meta-signature in PC based on 21 miRnome profiling studies. Four up-regulated miRNAs (hsa-miR-21-5p, hsa-miR-31-5p, hsa-miR-210-3p and hsa-miR-155-5p) and three down-regulated miRNAs (hsa-miR-217, hsa-miR-148a-3p and hsa-miR-375) were found to have significant diagnostic potential. Next, we investigated two independent cohorts from public databases, which included 136 tissues and 100 serum samples to validate the above findings. We obtained a relatively high clinical diagnostic accuracy of serum hsa-miR-21-5p in the diagnosis of PC. To extend the above findings, we further comprehensively evaluated the diagnostic values of circulating hsa-miR-21-5p based on 9 clinical investigations. As expected, our meta-analysis identified a remarkable clinical value of circulating hsa-miR-21-5p. Taken together, our data provided robust evidence for hsa-miR-21-5p as a diagnostic biomarker for PC patients.

Overwhelming evidence suggests that miRNAs play significant roles in the pathogenesis of multiple cancers including PC^51, 52^. However, miRNA expression profiling studies have always shown inconsistent results, which is primarily due to differences in measurement platforms and small sample sizes^14, 20^. To address this issue, systematic reviews and meta-analyses had been performed previously in order to identify miRNAs that were consistently reported among different studies. Unfortunately, no favorable results have been produced from such rigorous approaches, which is possibly due to a lack of cross-platform standardization of miRNA detection technologies and the unavailability of raw data. The RRA method is particularly suitable for the identification of miRNA meta-signatures, especially when input experiments are performed by varying technological platforms and when full rankings of miRNAs are not available. In the present study, we extracted 21 miRNA rank lists from 20 published studies that included a total of 905 PC and 449 noncancerous samples. After RRA analysis, we identified a meta-signature of four up-regulated (hsa-miR-21-5p, hsa-miR-31-5p, hsa-miR-210-3p and hsa-miR-155-5p) and three down-regulated miRNAs (hsa-miR-217, hsa-miR-148a-3p and hsa-miR-375). In 2013, Ma *et al*. reported a meta-signature consisting of 10 aberrantly expressed miRNAs, including seven up-regulated miRNAs (hsa-miR-155, hsa-miR-100, hsa-miR-21, hsa-miR-221, hsa-miR-31, hsa-miR-143 and hsa-miR-23a) and three down-regulated miRNAs (hsa-miR-217, hsa-miR-148a and hsa-miR-375)^20^. When our miRNA lists were compared with those in the study by Ma, our RRA analysis based on 21 studies added hsa-miR-210-3p as a significantly up-regulated miRNA, which was also confirmed by an independent validation study. A recent review by Hernandez *et al*.^53^ summarized the current knowledge of dysregulated miRNAs in PC and indicated that hsa-miR-210 was observed to be consistently up-regulated in different studies. All of the above results provided evidence for further clinical exploration of meta-signature miRNAs identified in our study.

Although pancreatic tissue biopsy remains the only gold standard for a definitive PC diagnosis, it causes discomfort and even pain during this invasive procedure. Therefore, non-invasive and accurate biomarkers are urgently needed. Currently, the only biomarker recommended by the National Comprehensive Cancer Network (NCCN) guidelines for PC diagnosis is carbohydrate antigen 19-9 (CA 19-9). However, CA 19-9 is also secreted by normal biliary epithelium, which limits its clinical utility because of its inadequate sensitivity and specificity. Very recently, Huang *et al*.^54^ summarized the diagnostic parameters of several classical biomarkers used in the detection of PC. The sensitivity and specificity of CA 19-9 were 0.75 and 0.82, respectively. Other serum biomarkers, such as CA-125, CEA, CA50, CA724, CA242 and AFP, also face the same limitations as those of CA 19-9 and do not have good clinical utility for the early diagnosis of PC. Recently, a diagnostic method based on the expression profile of miRNAs in body fluid samples, such as blood, saliva and digestive juice, has attracted considerable attention^55^. To extend the clinical application of meta-signature miRNAs in the diagnosis of PC, we first examined a validation cohort containing a total of 71 serum samples. Our data showed that circulating hsa-miR-21-5p had a significantly high diagnostic accuracy, with a sensitivity of 0.77 and a specificity of 0.80. Moreover, a meta-analysis of circulating hsa-miR-21-5p revealed a pooled sensitivity of 0.76 and a pooled specificity of 0.74. The use of circulating hsa-miR-21-5p alone or in combination with other biomarkers, such as CA 19-9, might benefit patients with PC.

In conclusion, a meta-signature in PC consisting of 7 aberrantly expressed miRNAs was statistically identified and clinically validated in our analysis. Among the meta-signature miRNAs, hsa-miR-21-5p was found to be highly prevalent in serum samples from PC patients. In the validation study, the diagnostic accuracy of serum hsa-miR-21-5p was found to be significantly higher than that of other miRNAs. Our meta-analysis further suggested that circulating hsa-miR-21-5p might be a promising biomarker for the diagnosis of PC. Further clinical studies that focus on these miRNAs, especially hsa-miR-21-5p, are needed to unveil their clinical significance in the diagnosis of PC.

## Electronic supplementary material

Supplementary tables and figures
