# Supplementary material for: Circulating miRNA-21-5p as a diagnostic biomarker for pancreatic cancer: evidence from comprehensive miRNA expression profiling analysis and clinical validation
Source: Sci Rep. 2017 May 10;7:1692. doi: 10.1038/s41598-017-01904-z (PMC5431820; doi:10.1038/s41598-017-01904-z)
Supplement: Supplementary file 1 — Supplementary tables and figures [file 41598_2017_1904_MOESM1_ESM.pdf]

Circulating miRNA-21-5p as a diagnostic biomarker for pancreatic cancer: evidence from comprehensive miRNA expression profiling analysis and clinical validation

Running title: *Circulating miRNA-21-5p in PC diagnosis*

Kai Qu [1,#], Xing Zhang [1,#], Ting Lin [1,#], Tian Liu [2], Zhixin Wang [3], Sushun Liu [4], Lei Zhou [5], Jichao Wei [6], Hulin Chang [7], Ke Li [8], Zheng Wang [1], Chang Liu [1,*], Zheng Wu [1,*]

For correspondence:

Zheng Wu, Ph.D., M.D.,

Address: Department of Hepatobiliary Surgery, The First Affiliated Hospital of Xi'an Jiaotong University, Xi'an 710061, China. Email: wuzheng@126.com. Telephone: +86-29-85323895. Fax number: +86-29-85324695.

or **Chang Liu,** Ph.D., M.D.,

Address: Department of Hepatobiliary Surgery, The First Affiliated Hospital of Xi'an Jiaotong University, Xi'an 710061, China. Email: liuchangdoctor@163.com. Telephone: +86-29-85323900. Fax number: +86-29-85324695.

Supplementary Figure Legends

Figure S1 Flow diagram of search strategy and study selection for RRA analysis.

Figure S2 Kaplan–Meier analysis of overall survival for patients with different meta-signature miRNA expression levels.

Figure S3 Expression correlation between serum and tissue has-miR-21-5p.

Figure S4 Flow diagram of search strategy and study selection for diagnostic meta-analysis.

Figure S5 Sensitivity analysis of miR-21-5p in PC. (A) goodness-of-fit; (B) bivariate normality; (C) influence analysis; (D) outlier detection.

Figure S6 Funnel graph for the assessment of potential publication bias for studies of miR-21-5p.

Figure S7 Forest plots showing the sensitivity and specificity of miR-21-5p in Asian subgroup. (A) Forest plot showing the sensitivity of miR-21-5p in the diagnosis of PC. (B) Forest plot showing the specificity of miR-21-5p in the diagnosis of PC.

Figure S8 Forest plots showing the sensitivity and specificity of miR-21-5p in blood sample subgroup. (A) Forest plot showing the sensitivity of miR-21-5p in the diagnosis of PC. (B) Forest plot showing the specificity of miR-21-5p in the diagnosis of PC.

Figure S9 Forest plots showing the sensitivity and specificity of miR-21-5p in healthy volunteer subgroup. (A) Forest plot showing the sensitivity of miR-21-5p in the diagnosis of PC. (B) Forest plot showing the specificity of

miR-21-5p in the diagnosis of PC.

Figure S10 Forest plots showing the sensitivity and specificity of miR-21-5p in patients with benign diseases. (A) Forest plot showing the sensitivity of miR-21-5p in the diagnosis of PC. (B) Forest plot showing the specificity of miR-21-5p in the diagnosis of PC.

PRISMA 2009 Flow Diagram

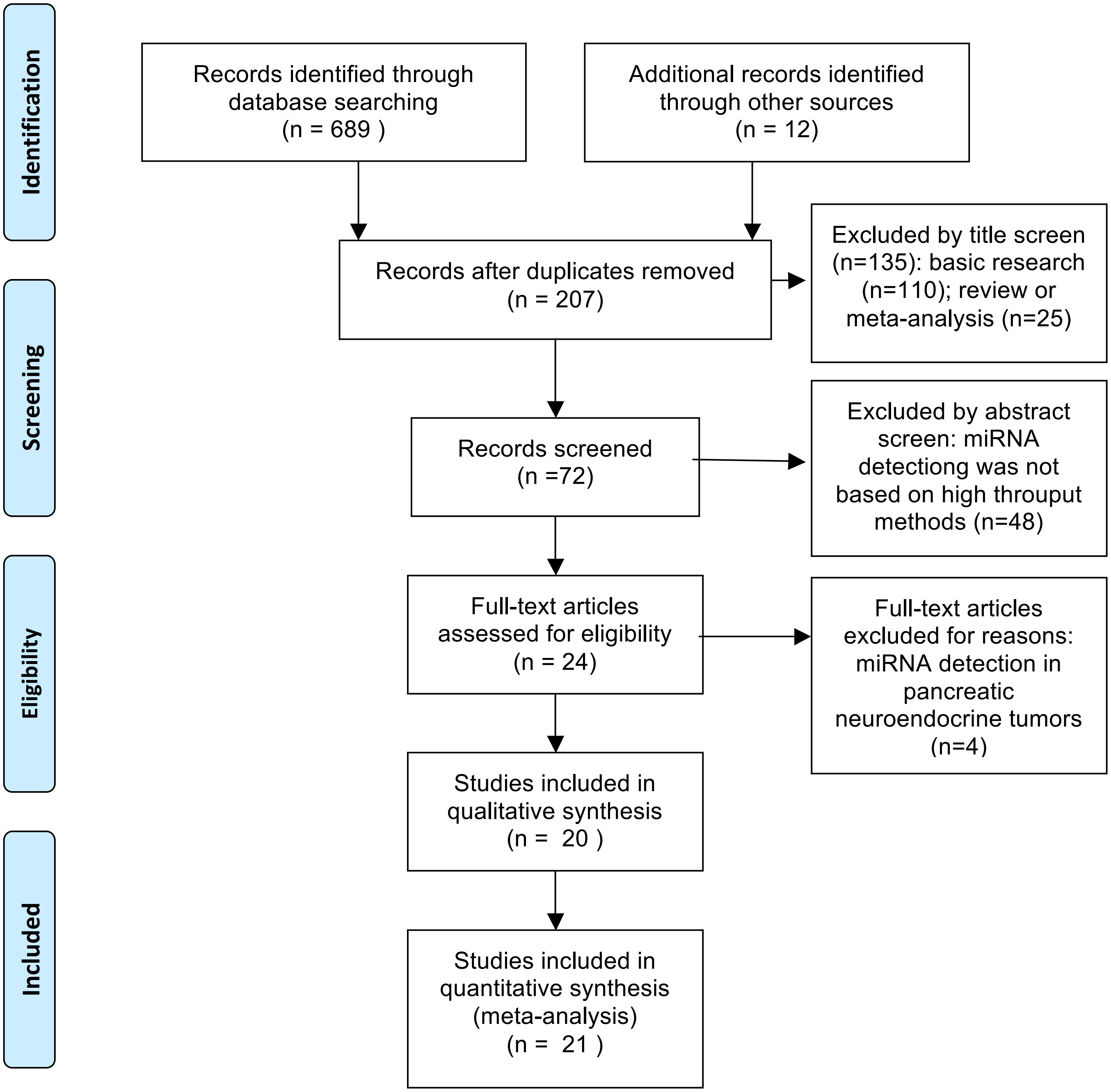

From: Moher D, Liberati A, Tetzlaff J, Altman DG, The PRISMA Group (2009). *P*referred *R*eporting *I*tems for *S*ystematic Reviews and *M*eta-*A*nalyses: The PRISMA Statement. PLoS Med 6(6): e1000097. doi:10.1371/journal.pmed1000097

For more information, visit www.prisma-statement.org.

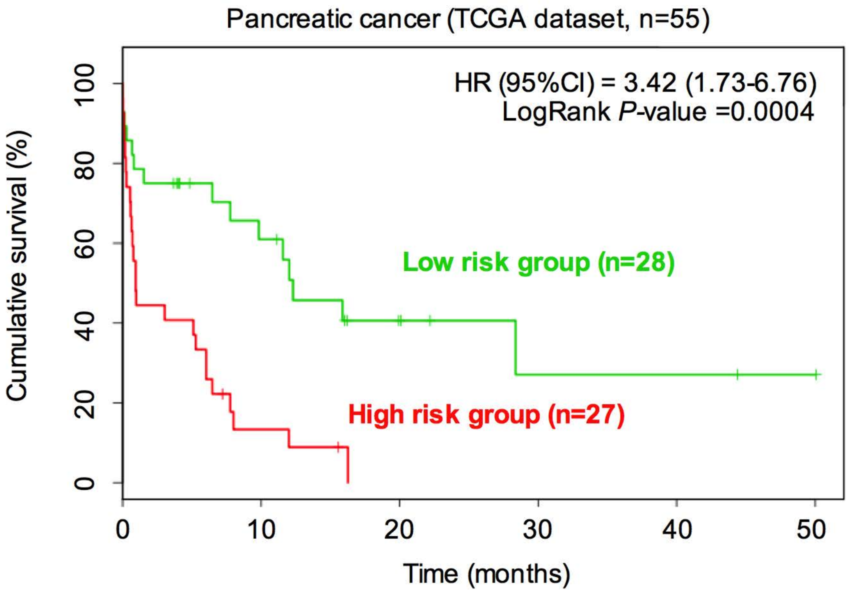

Pancreatic cancer (TCGA dataset, n=55)
HR (95%CI) = 3.42 (1.73-6.76)
LogRank P-value =0.0004
Low risk group (n=28)
High risk group (n=27)
Cumulative survival (%)
Time (months)
0
20
40
60
80
100
0
10
20
30
40
50

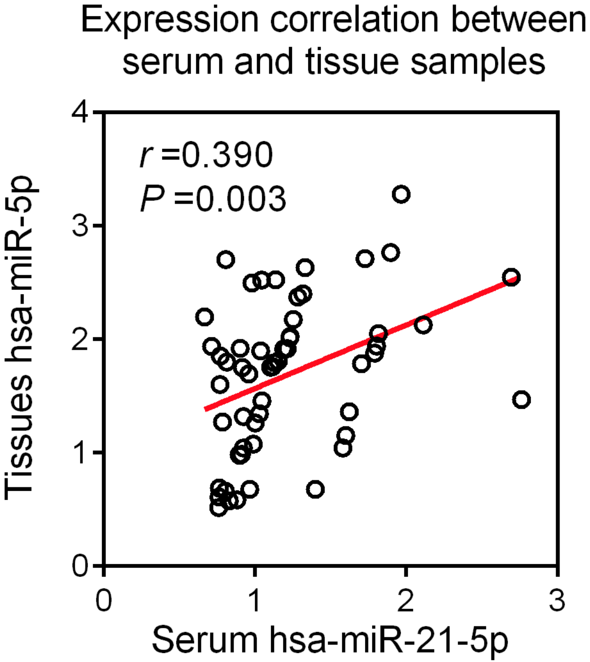

Expression correlation between
serum and tissue samples
r =0.390
P =0.003
Tissues hsa-miR-5p
Serum hsa-miR-21-5p
0
1
2
3
4

PRISMA 2009 Flow Diagram

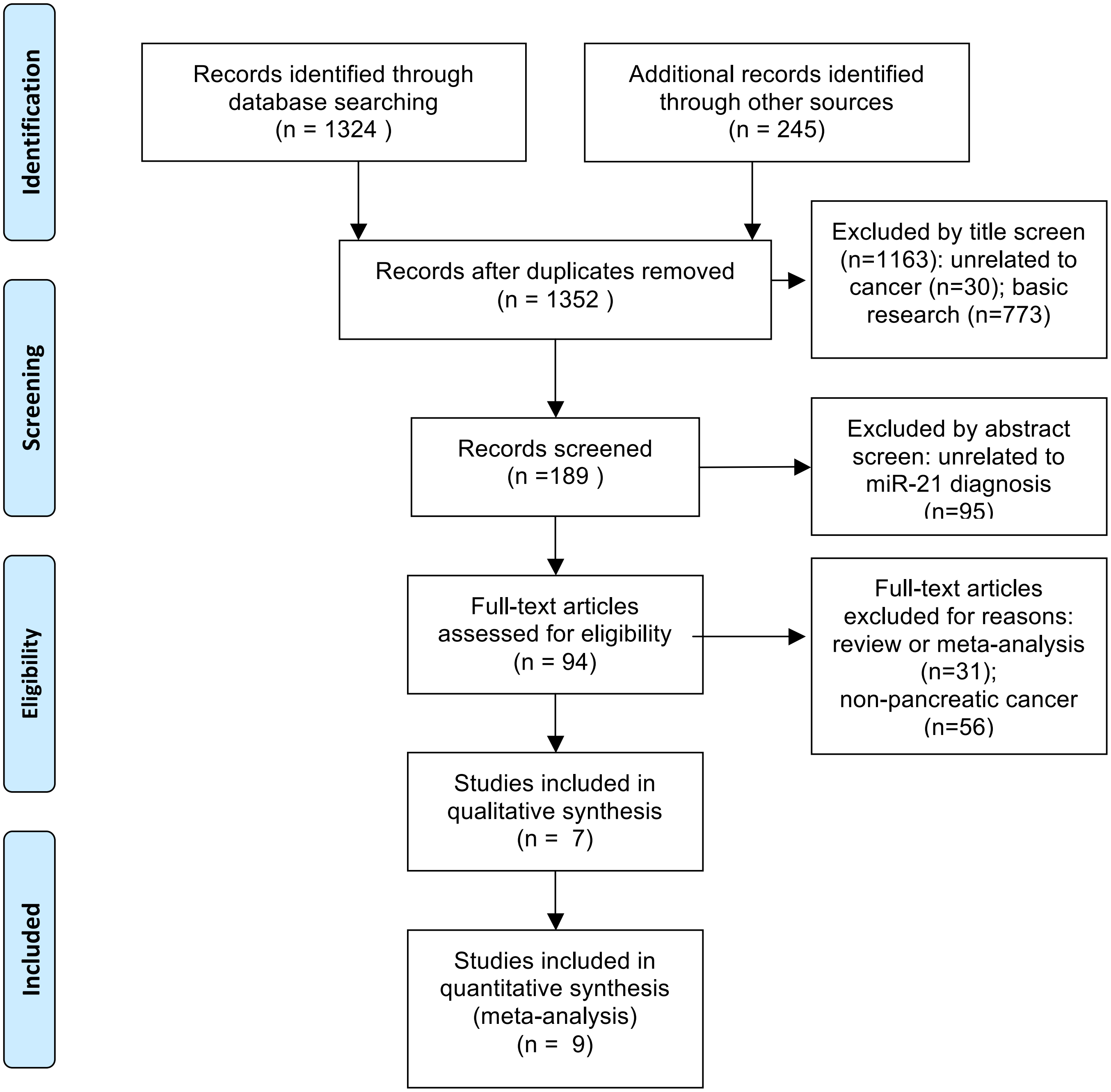

From: Moher D, Liberati A, Tetzlaff J, Altman DG, The PRISMA Group (2009). *P*referred *R*eporting *I*tems for *S*ystematic Reviews and *M*eta-*A*nalyses: The PRISMA Statement. PLoS Med 6(6): e1000097. doi:10.1371/journal.pmed1000097

For more information, visit www.prisma-statement.org.

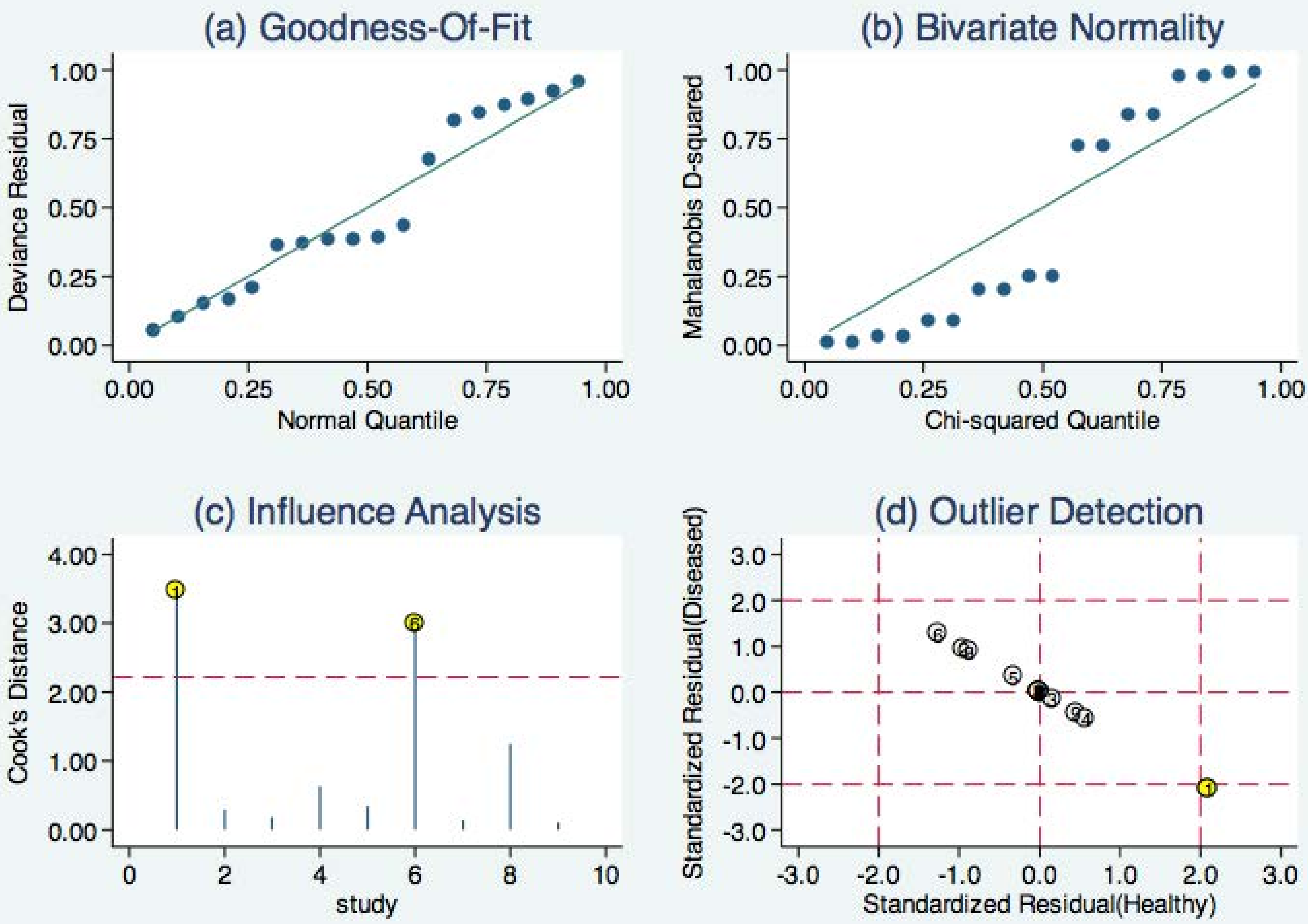

(a) Goodness-Of-Fit
Deviance Residual
Normal Quantile
0.00
0.25
0.50
0.75
1.00
(b) Bivariate Normality
Mahalanobis D-squared
Chi-squared Quantile
(c) Influence Analysis
Cook's Distance
study
0
2
4
6
8
10
0.00
1.00
2.00
3.00
4.00
(d) Outlier Detection
Standardized Residual(Diseased)
Standardized Residual(Healthy)
-3.0
-2.0
-1.0
0.0
1.0
2.0
3.0

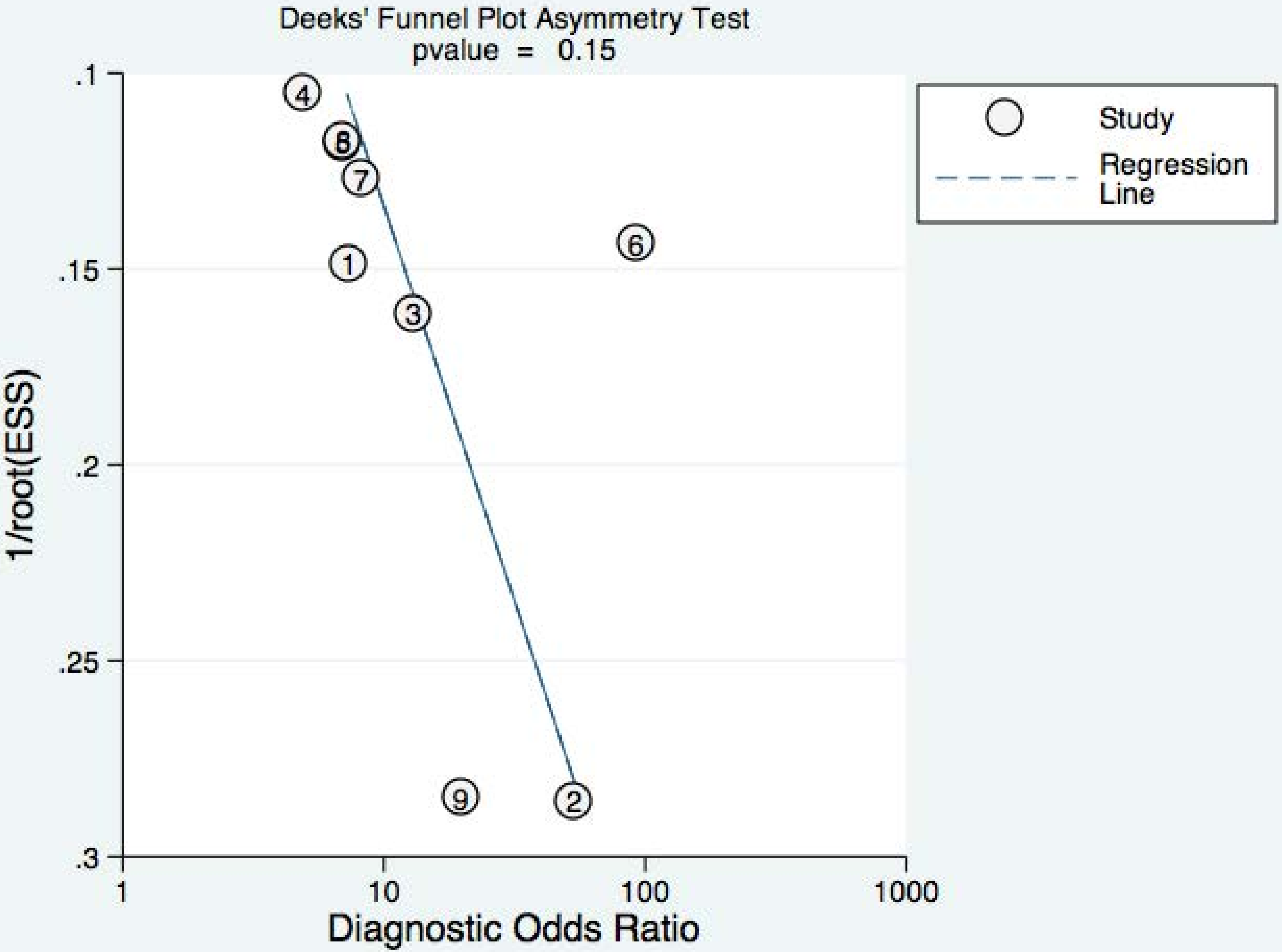

Deeks' Funnel Plot Asymmetry Test
pvalue = 0.15
1/root(ESS)
.1
.15
.2
.25
.3
Diagnostic Odds Ratio
1
10
100
1000
Study
Regression Line

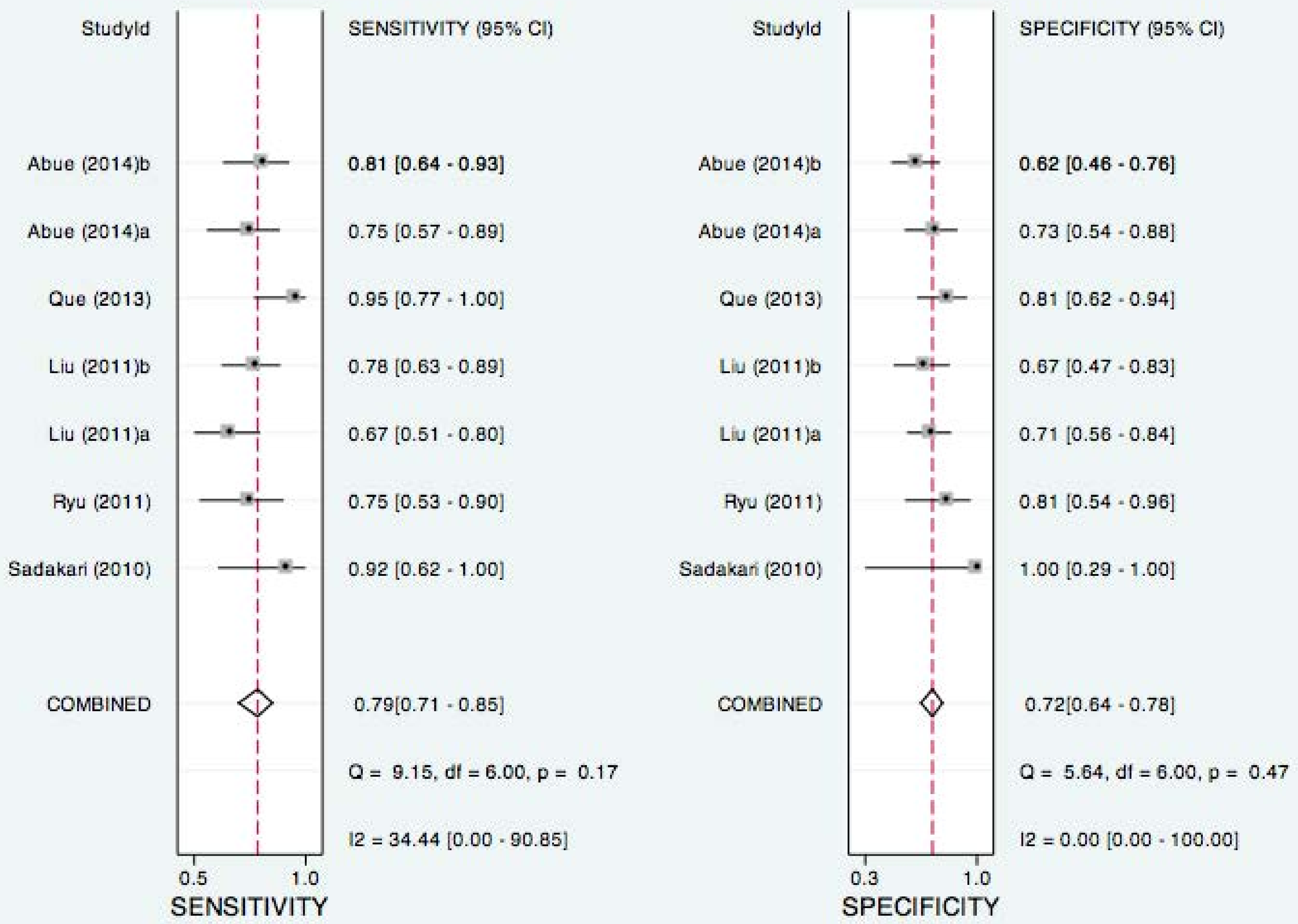
StudyId
SENSITIVITY (95% CI)
Abue (2014)b
0.81 [0.64 - 0.93]
Abue (2014)a
0.75 [0.57 - 0.89]
Que (2013)
0.95 [0.77 - 1.00]
Liu (2011)b
0.78 [0.63 - 0.89]
Liu (2011)a
0.67 [0.51 - 0.80]
Ryu (2011)
0.75 [0.53 - 0.90]
Sadakari (2010)
0.92 [0.62 - 1.00]
COMBINED
0.79[0.71 - 0.85]
Q = 9.15, df = 6.00, p = 0.17
I2 = 34.44 [0.00 - 90.85]
0.5
1.0
SENSITIVITY
StudyId
SPECIFICITY (95% CI)
Abue (2014)b
0.62 [0.46 - 0.76]
Abue (2014)a
0.73 [0.54 - 0.88]
Que (2013)
0.81 [0.62 - 0.94]
Liu (2011)b
0.67 [0.47 - 0.83]
Liu (2011)a
0.71 [0.56 - 0.84]
Ryu (2011)
0.81 [0.54 - 0.96]
Sadakari (2010)
1.00 [0.29 - 1.00]
COMBINED
0.72[0.64 - 0.78]
Q = 5.64, df = 6.00, p = 0.47
I2 = 0.00 [0.00 - 100.00]
0.3
1.0
SPECIFICITY

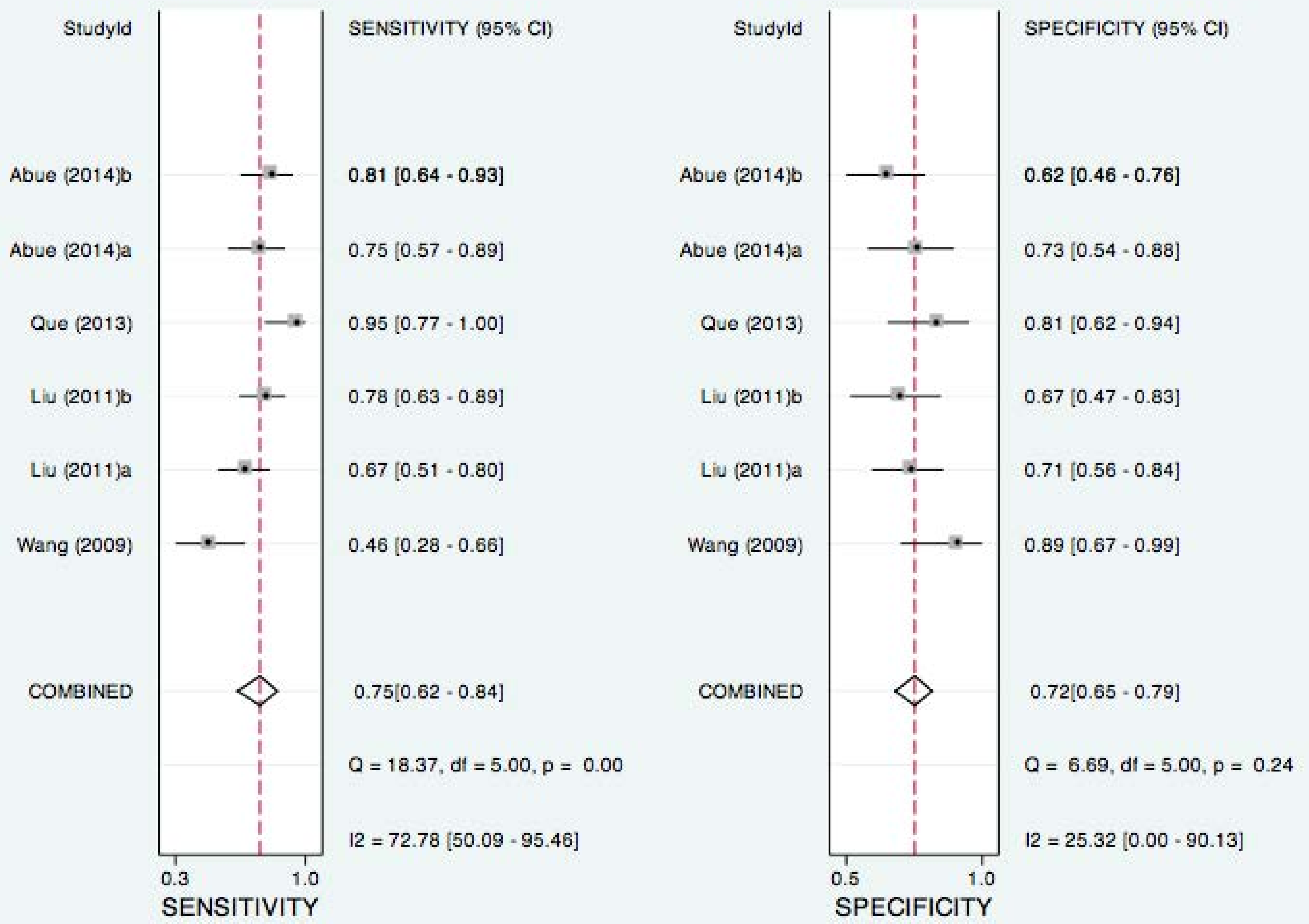
StudyId
SENSITIVITY (95% CI)
Abue (2014)b
0.81 [0.64 - 0.93]
Abue (2014)a
0.75 [0.57 - 0.89]
Que (2013)
0.95 [0.77 - 1.00]
Liu (2011)b
0.78 [0.63 - 0.89]
Liu (2011)a
0.67 [0.51 - 0.80]
Wang (2009)
0.46 [0.28 - 0.66]
COMBINED
0.75[0.62 - 0.84]
Q = 18.37, df = 5.00, p = 0.00
I2 = 72.78 [50.09 - 95.46]
0.3
1.0
SENSITIVITY
StudyId
SPECIFICITY (95% CI)
Abue (2014)b
0.62 [0.46 - 0.76]
Abue (2014)a
0.73 [0.54 - 0.88]
Que (2013)
0.81 [0.62 - 0.94]
Liu (2011)b
0.67 [0.47 - 0.83]
Liu (2011)a
0.71 [0.56 - 0.84]
Wang (2009)
0.89 [0.67 - 0.99]
COMBINED
0.72[0.65 - 0.79]
Q = 6.69, df = 5.00, p = 0.24
I2 = 25.32 [0.00 - 90.13]
0.5
1.0
SPECIFICITY

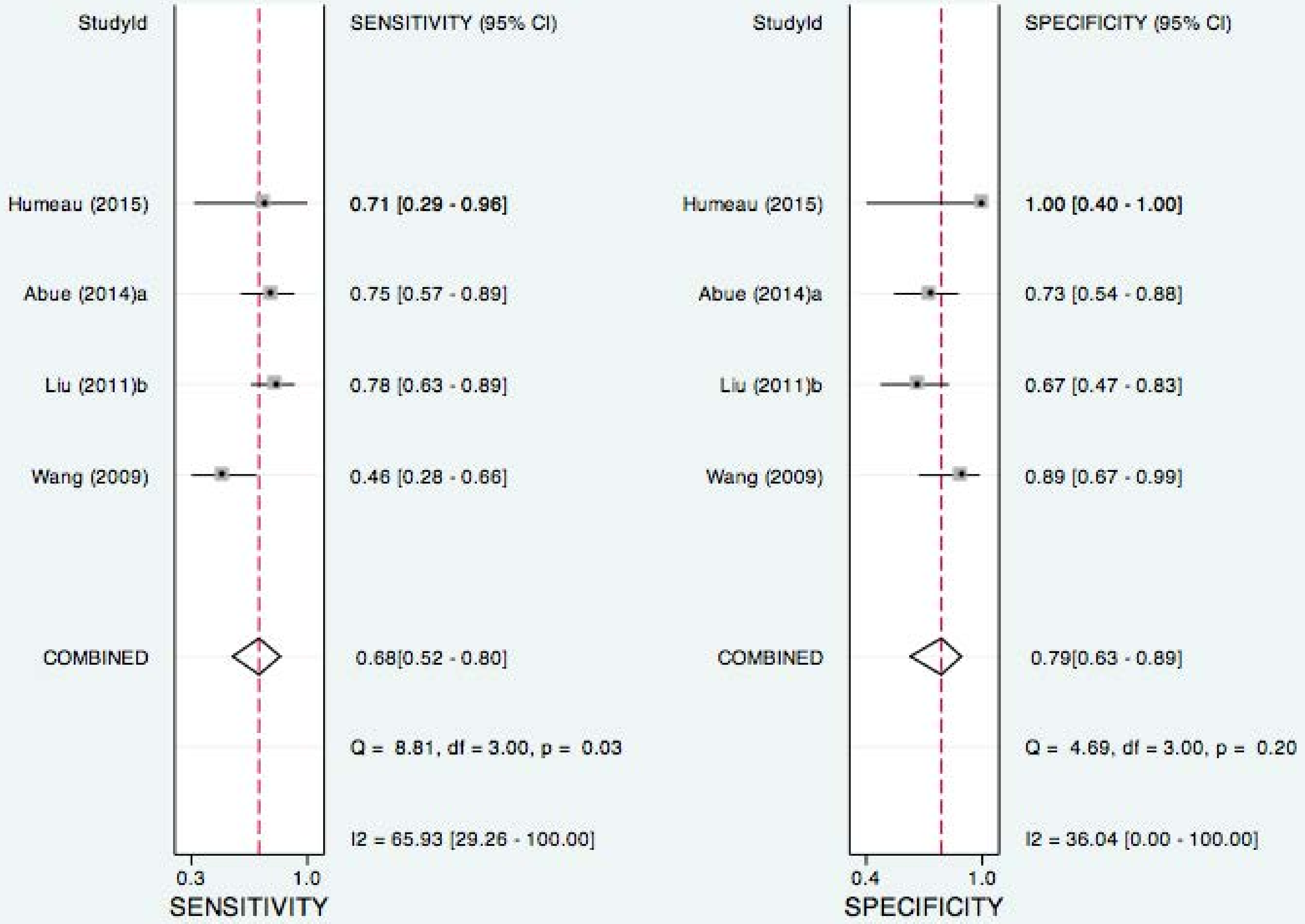

StudyId
SENSITIVITY (95% CI)
Humeau (2015)
0.71 [0.29 - 0.96]
Abue (2014)a
0.75 [0.57 - 0.89]
Liu (2011)b
0.78 [0.63 - 0.89]
Wang (2009)
0.46 [0.28 - 0.66]
COMBINED
0.68[0.52 - 0.80]
Q = 8.81, df = 3.00, p = 0.03
I2 = 65.93 [29.26 - 100.00]
0.3
1.0
SENSITIVITY
StudyId
SPECIFICITY (95% CI)
Humeau (2015)
1.00 [0.40 - 1.00]
Abue (2014)a
0.73 [0.54 - 0.88]
Liu (2011)b
0.67 [0.47 - 0.83]
Wang (2009)
0.89 [0.67 - 0.99]
COMBINED
0.79[0.63 - 0.89]
Q = 4.69, df = 3.00, p = 0.20
I2 = 36.04 [0.00 - 100.00]
0.4
1.0
SPECIFICITY

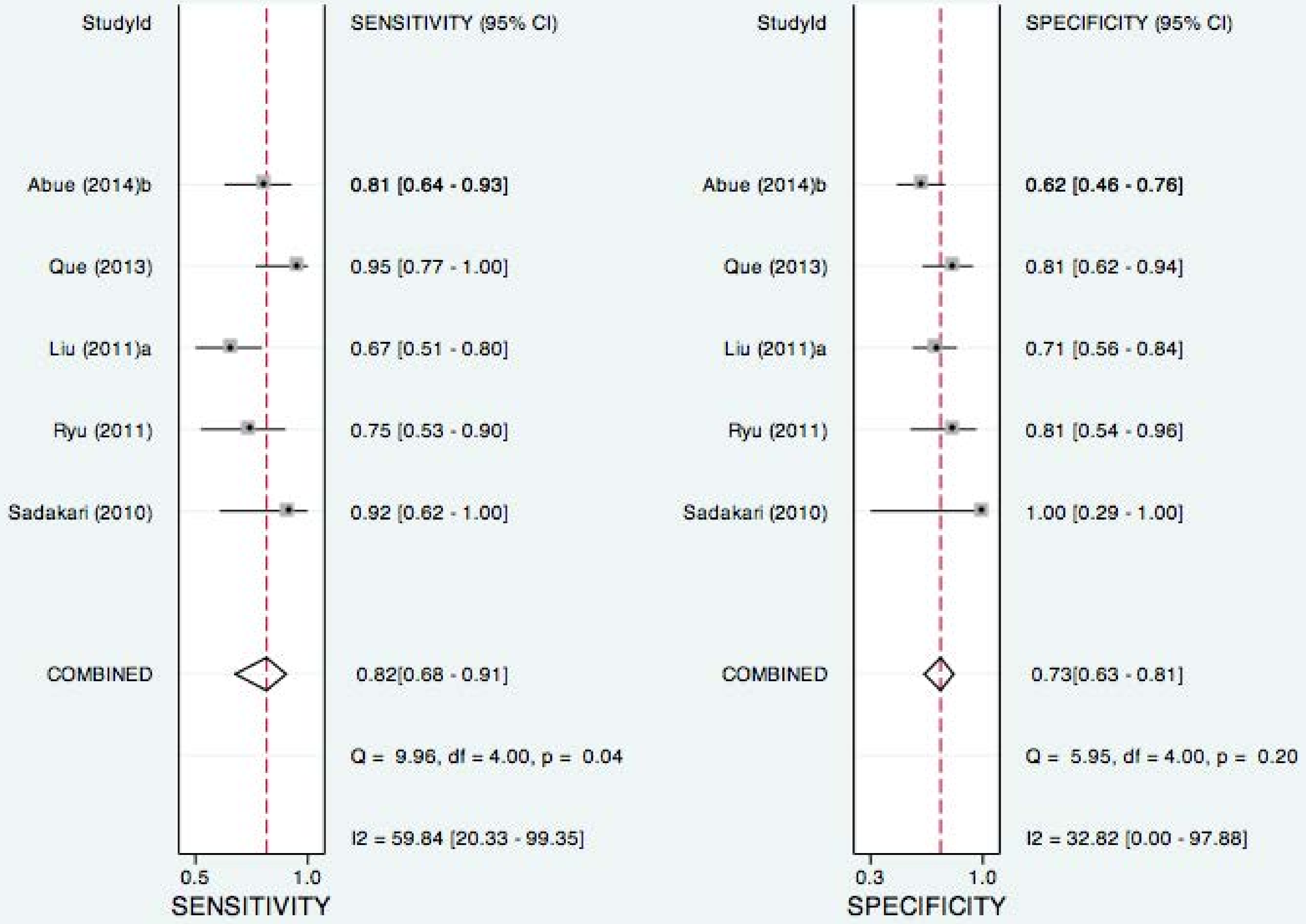
StudyId
SENSITIVITY (95% CI)
Abue (2014)b
0.81 [0.64 - 0.93]
Que (2013)
0.95 [0.77 - 1.00]
Liu (2011)a
0.67 [0.51 - 0.80]
Ryu (2011)
0.75 [0.53 - 0.90]
Sadakari (2010)
0.92 [0.62 - 1.00]
COMBINED
0.82[0.68 - 0.91]
Q = 9.96, df = 4.00, p = 0.04
I2 = 59.84 [20.33 - 99.35]
0.5
1.0
SENSITIVITY
StudyId
SPECIFICITY (95% CI)
Abue (2014)b
0.62 [0.46 - 0.76]
Que (2013)
0.81 [0.62 - 0.94]
Liu (2011)a
0.71 [0.56 - 0.84]
Ryu (2011)
0.81 [0.54 - 0.96]
Sadakari (2010)
1.00 [0.29 - 1.00]
COMBINED
0.73[0.63 - 0.81]
Q = 5.95, df = 4.00, p = 0.20
I2 = 32.82 [0.00 - 97.88]
0.3
1.0
SPECIFICITY

Table S1 Clinicopathological characteristics of patients in validation groups

Variables	
Age, median (range), yr	52 (45-71)[a]
Gender	
Male	58 (61.1%)
Female	37 (38.9%)
Tumor size	
≥ 2cm	50 (52.6%)
< 2cm	45 (47.4%)
Histology	
Poorly differentiated	47 (49.5%)
Well or moderately differentiated	48 (50.5%)
Tumor Grade (UICC)	
Stage I-IIA	41 (43.2%)
Stage IIB-IV	54 (56.8%)
Resection margin status	
R0	41 (43.2%)
R1	54 (56.8%)

a. Data is presented as mean and range

Table S2 Characteristics of analyzed datasets

Author (year)	Country	Ethnicity	Period	No. of sample (T/N)	Tissue type	Control source	High-throughput method	Total miRNAs	Cut-off criteria
Bloomston (2007)	USA	Caucasian	2000.01-2005.12	65/42	Frozen tissue	Chronic pancreatitis tissues	Custom miRNA microarray chips	326	FC>2
Lee (2007)	USA	Caucasian	NA	28/6	Frozen tissue	Normal pancreas tissues	qRT-PCR using TaqMan microRNA Assays	222	p<0.01
Szafranska (2007)	Germany	Caucasian	NA	10/7	Frozen tissue	Normal pancreas tissues	Custom miRNA microarray chips	281	p<0.05
Zhang (2009)	USA	Caucasian	NA	17/18	Frozen tissue	Adjacent non-tumorous tissues	qRT-PCR using QuantiMir System	95	FC>3.3
Hao (2011)	China	Asian	2009-2011	40/40	Frozen tissue	Adjacent non-tumorous tissues	qRT-PCR using TaqMan microRNA Assays	71	p<0.05
Zhang (2011)	China	Asian	2009-2012	20/20	Frozen tissue	Adjacent non-tumorous tissues	miRCURY LNA human microRNA Array	1200	p<0.05
Ali (2012)	USA	Caucasian	NA	15/15	FFPE tissue	Adjacent non-tumorous tissues	qRT-PCR using SYBR Green miRNA-based assay	228	NA
Bauer (2012)	Germany	Caucasian	NA	94/16	Frozen tissue	Chronic pancreatitis tissues	Geniom biochip miRNA homo sapiens	863	p<0.05
Jamieson (2012)	UK	Caucasian	NA	48/10	Frozen tissue	Adjacent non-tumorous tissues	Agilent's Human miRNA Microarray	723	p<0.001
Munding (2012)	Germany	Caucasian	NA	57/48	FFPE tissue	Chronic pancreatitis tissues	Agilent Human microRNA Microarray v2.0	719	p<0.05
Nagao (2012)	Japan	Asian	NA	65/65	Frozen tissue	Adjacent non-tumorous tissues	3D-Gene miRNA microarray platform and qRT-PCR using TaqMan microRNA Assays	900	FC>2
Piepoli (2012)	Italy	Caucasian	NA	17/17	Frozen tissue	Adjacent non-tumorous tissues	GeneChip miRNA Array	846	p<0.01
Schultz (2012)	Denmark	Caucasian	1976-2008	160/51	Frozen tissue	Chronic pancreatitis (23) and normal pancreas tissues (28)	TaqMans Array Human MicroRNA A+B Cards v2.0	664	p<0.05
Yu (2012)a	USA	Caucasian	NA	34/15	Frozen tissue	Normal pancreas tissues	TaqMans Array Human MicroRNA A+B Cards	735	FC>2
Yu (2012)b	USA	Caucasian	NA	11/15	Frozen tissue	Normal pancreas tissues	TaqMans Array Human MicroRNA A+B Cards	735	FC>2
Lubezky (2013)	Israel	Caucasian	2002.02-2011	45/10	Frozen tissue	Normal pancreas tissues	GeneChip miRNA Array	846	p<0.05
Zhao (2013)	China	Asian	2008.01-2010.06	10/10	Frozen tissue	Adjacent non-tumorous tissues	Agilent Human microRNA Microarray	713	p<0.05

Collins 2014)	USA	Caucasian	1993-2007	9/9	Frozen tissue	Adjacent non-tumorous tissues	nanoString nCounter platform	326	p<0.01
Hong (2014)	Korea	Asian	2013.01-2013.08	11/11	Frozen tissue	Adjacent non-tumorous tissues	miRCURY LNA human microRNA Array	3100	p<0.05
Zhang (2014)	China	Asian	NA	9/3	Frozen tissue	Adjacent non-tumorous tissues	Agilent Human microRNA Microarray	713	p<0.05
Kojima (2015)	Japan	Asian	2010-2012	100/21	Frozen tissue	Normal pancreas tissues	3D-Gene miRNA microarray platform	2555	p<0.01

FFPE, formalin-fixed paraffin-embedded; FC, fold change; NA, not available.

Table S3 Validated targets of seven differentially expressed miRNAs in pancreatic cancer.

**miR-21-5p**	**miR-31-5p**	**miR-210-3p**	**miR-155-5p**	**miR-217**	**miR-148a-3p**	**miR-375**
RASGRP1	*RHOA*	*HOXA9*	*UQCRFS1*	*SIRT1*	*DNMT1*	*TIMM8A*
CDC25A	*PPP2R2A*	*TP53I11*	*MEIS1*	*ROBO1*	*HLA-G*	*MTPN*
BCL2	*LATS2*	*PIM1*	*TAB2*	*PTEN*	*TGIF2*	*PDK1*
TM9SF3	*SATB2*	*HOXA1*	*MECP2*	*FHIT*	*DNMT3B*	*USP1*
RTN4	*FOXP3*	*FGFRL1*	*SOCS1*	*KRAS*	*NR1I2*	*JAK2*
PLOD3	*WASF3*	*RAD52*	*MSH6*	*NR4A2*	*RPS6KA5*	*ADIPOR2*
NCAPG	*YY1*	*NPTX1*	*MSH2*	*EZH2*	*CCKBR*	*C1QBP*
DERL1	*RET*	*EFNA3*	*MLH1*	*TRAPPC2P1*	*IRS1*	*YAP1*
BASP1	*NUMB*	*CASP8AP2*	*INPP5D*	*PPM1D*	*ACVR1*	*PLAG1*
JAG1	*NFAT5*	*PTPN1*	*DET1*	*SMAD7*	*BCL2*	*MTDH*
REST	*KLF13*	*BDNF*	*SMAD5*	*E2F3*	*TMED7*	*RASD1*
SMARCA4	*JAZF1*	*XIST*	*HIVEP2*	*DACH1*	*PBXIP1*	*YY1AP1*
SPRY2	*HOXC13*	*CPEB2*	*ZNF652*	*FOXO3*	*CDC25B*	*ELAVL4*
DUSP10	*ETS1*	*GPD1L*	*ZIC3*	*GPC5*	*MMP7*	*SP1*
TIMP3	*ITGA5*	*ISCU*	*BACH1*	*SIRT1*	*WNT10B*	*MAP3K8*
SOX5	*MPRIP*	*NCAM1*	*Arntl*		*CDKN1B*	*FZD8*
MTAP	*MMP16*	*E2F3*	*Sla*		*SERPINE1*	*LDHB*
DOCK7	*RDX*	*DDAH1*	*Hif1a*		*ITGB8*	*YWHAZ*
DOCK5	*CXCL12*	*MNT*	*Csf1r*		*VAV2*	*KIAA1524*
RECK	*ARPC5*	*AIFM3*	*Jarid2*		*ITGA5*	*TP53*
PIAS3	*FZD3*	*VMP1*	*Cebpb*		*ROCK1*	*ERBB2*
TGFBR2	*DMD*	*TFRC*	*Picalm*		*SMAD2*	*IGF1R*
PTEN	*TIAM1*	*HSD17B1*	*CSNK1A1*		*MET*	*PIK3CA*
E2F1	*ICAM1*	*NDUFA4*	*APC*		*BCL2L11*	*PHLPP1*
TGFBI	*DKK1*	*SDHD*	*TRIP13*		*MAFB*	*RHOA*
LRRFIP1	*DACT3*	*STMN1*	*TBCA*		*ERRFI1*	*KCNQ2*
MARCKS	*PRKCE*	*DIMT1L*	*SMAD1*		*S1PR1*	*TIMM8A*
SP1	*RASA1*	*ALDH5A1*	*SDCBP*		*USP4*	*BCL2L11*
CCL20	*STK40*	*FOXN3*	*RHEB*		*DNMT1*	*RAB10*
TPM1	*MCM2*	*MCM3*	*POLE3*		*RUNX3*	*PARP1*
NFIB	*CDK1*	*IGFBP3*	*PKN2*		*MYC*	*CAB39*
APAF1	*CREG1*	*COL4A2*	*PHC2*			*DLG4*
BTG2	*MLH1*	*INPP5A*	*NARS*			*ITGB1*
PDCD4	*MET*	*EHD2*	*MYO10*			*SETD8*
RHOB	*SRC*	*SH3BGRL*	*DHX40*			*CASP3*
ANP32A	*MAP4K4*	*PTPN2*	*CLDN1*			*CDC42*
SERPINB5	*RAB27A*	*LDHA*	*ARID2*			*EIF2AK2*
BMPR2	*TBXA2R*	*LDHB*	*ARFIP1*			*BCL2*

RASA1	*C11orf30*	*FOXP3*	*TM6SF1*			*NCAM1*
NCOA3	*ARID1A*	*HIF3A*	*MATR3*			*LEPROTL1*
PCBP1	*ABCB9*	*BNIP3*	*LDOC1*			
JMY	*RHOBTB1*	*KCMF1*	*PHF17*			
TOPORS	*SP7*	*P4HB*	*RHOA*			
HNRNPK	*HIF1AN*	*MRE11A*	*AGTR1*			
DAXX	*RHOA*	*XPA*	*PKIA*			
TP53BP2	*SELE*	*ROD1*	*RNF123*			
TP63		*HIF1A*	*TP53INP1*			
TGFBR3		*SMCHD1*	*IKBKE*			
PPIF		*TNPO1*	*FGF7*			
MSH2		*CBX1*	*KDM3A*			
MSH6		*ABCB9*	*NFATC2IP*			
TIAM1		*CDK10*	*SPI1*			
ISCU		*DENND6A*	*Cux1*			
MEF2C		*HOXA3*	*Sfpi1*			
EIF4A2		*KIAA1161*	*EDN1*			
ANKRD46		*MDGA1*	*FOXO3*			
EGFR		*MID1IP1*	*MAFB*			
IL1B		*SEH1L*	*TSHZ3*			
ICAM1		*UBQLN1*	*RUNX2*			
PLAT		*SERTAD2*	*JUN*			
PTX3		*ACVR1B*	*IFNGR1*			
TNFAIP3		*APC*	*KBTBD2*			
CCR1		*ATP11C*	*KRAS*			
CDK2AP1		*CHD9*	*ETS1*			
DOCK4		*CLASP2*	*TLE4*			
PPARA		*ELK3*	*CYR61*			
NTF3		*PTAR1*	*ICAM1*			
COL4A1		*NIPBL*	*SELE*			
FASLG		*MIB1*	*SMAD2*			
SOD3		*HECTD1*	*MYB*			
TCF21			*SKI*			
SMAD7			*GCSAM*			
SERPINI1			*CKAP5*			
BCL6			*SOX6*			
SMN1			*IL13RA1*			
HPGD			*FADD*			
MYD88			*BCL6*			
IRAK1			*MITF*			
VHL			*MAP3K10*			

GDF5			*NOS3*			
MAP2K3			*ANAPC16*			
SETD2			*C17orf80*			
CLU			*MYLK*			
TNFRSF10B			*PSIP1*			
IGF1R			*GCFC2*			
RPS7			*EXOSC2*			
FMOD			*LNX2*			
TGIF1			*ZNF248*			
ERBB2			*CHD9*			
VEGFA			*MEF2A*			
AKT2			*CAB39*			
YOD1			*CLUAP1*			
STAT3			*CARD11*			
SATB1			*PCDH9*			
WWP1			*ZNF561*			
NFIA			*CARHSP1*			
SIRT2			*C16orf62*			
GAS5			*LIN7C*			
RHO			*CBR4*			
E2F2			*GPM6B*			
HIPK3			*LRIF1*			
MYC			*TAF5L*			
SOX2			*HERC4*			
RMND5A			*MORC3*			
MMP2			*MBNL3*			
SASH1			*UPF2*			
MMP9			*TSPAN14*			
ELAVL4			*INTS6*			
TOR1AIP2			*YWHAZ*			
PELI1			*PRKAR1A*			
IL12A			*SSX2IP*			
SECISBP2L			*FAM199X*			
RFFL			*RAC1*			
CXCL10			*PLS1*			
			SAP30L			
			MRPS27			
			CEP41			
			CIAPIN1			
			CCDC82			
			ACTR2			

			TRAK1			
			CYP2U1			
			SLC35F2			
			ZNF493			
			HAL			
			IL17RB			
			TBC1D14			
			ZNF254			
			GABARAPL1			
			IGJ			
			RAPGEF2			
			WBP1L			
			PBRM1			
			MRPL18			
			MAP3K14			
			ARMC2			
			LCORL			
			APAF1			
			MPP5			
			RAB11FIP2			
			NOVA1			
			RBAK			
			ARL15			
			MYO1D			
			LRRC59			
			ANXA2			
			TTF1			
			FAM91A1			
			CCDC41			
			KIAA0430			
			CDC40			
			DCUN1D2			
			KLHL5			
			AGO4			
			HBP1			
			WWC1			
			WEE1			
			GOLT1B			
			PALD1			
			CCND1			
			ZNF83			

			PHF14			
			TBC1D8B			
			INPP5F			
			ARPC3			
			KRCC1			
			FAM177A1			
			UBTD2			
			SECISBP2			
			PAK2			
			SLC33A1			
			ZNF28			
			MCM8			
			SMARCA4			
			TCF12			
			TOMM20			
			UBQLN1			
			VPS18			
			WHSC1L1			
			MASTL			
			MYBL1			
			GATM			
			E2F2			
			FAM135A			
			C3orf18			
			ARL6IP5			
			SHANK2			
			SH3PXD2A			
			PIK3R1			
			VHL			
			MMP16			
			MYC			
			SEL1L			
			DOCK1			
			RAD51			
			THRB			
			TERF1			
			MXI1			
			UQCRFS1			
			PDLIM5			
			MSI2			
			BRPF3			

			FLI1			
			MYD88			
			NKX3-1			
			OLR1			
			SMAD4			
			CD68			
			SMAD3			
			IL8			
			ZNF611			
			TRIM32			
			PPL			
			DNAJC19			
			TWF1			
			FLT1			
			VCAM1			
			INADL			
			ITK			
			IL2			
			ZNF431			
			WNK1			
			ZNF714			
			ETNK2			
			TRPS1			
			PEA15			
			PTN			
			SOCS3			

Table S4 The combinatorial effect of 7 miRNAs in KEGG pathways

#	KEGG pathway	FDR	#Targets
1	Colorectal cancer	5.14E-18	22
2	Pathways in cancer	1.51E-16	64
3	Hepatitis B	2.74E-13	33
4	Prion diseases	1.97E-12	3
5	Pancreatic cancer	9.12E-12	22
6	Bladder cancer	2.05E-08	13
7	Endometrial cancer	3.51E-08	14
8	p53 signaling pathway	3.65E-08	18
9	Protein processing in endoplasmic reticulum	4.67E-08	34
10	RNA transport	7.75E-08	31
11	Adherens junction	2.22E-07	18
12	Prostate cancer	8.49E-07	20
13	TGF-beta signaling pathway	1.06E-06	14
14	mTOR signaling pathway	1.68E-06	16
15	Glycosaminoglycan biosynthesis - chondroitin sulfate	9.11E-06	3
16	Small cell lung cancer	9.11E-06	18
17	Non-small cell lung cancer	6.53E-05	13
18	Focal adhesion	1.39E-04	32
19	PI3K-Akt signaling pathway	1.70E-04	49
20	Arrhythmogenic right ventricular cardiomyopathy (ARVC)	1.89E-04	13
21	Melanoma	3.12E-04	14
22	Chronic myeloid leukemia	3.34E-04	15
23	Apoptosis	1.21E-03	16
24	Insulin signaling pathway	1.49E-03	22
25	Steroid biosynthesis	2.15E-03	4
26	Sphingolipid metabolism	2.15E-03	11
27	Malaria	2.36E-03	10
28	Regulation of actin cytoskeleton	3.96E-03	32
29	Cell cycle	4.22E-03	23
30	Viral myocarditis	5.98E-03	12
31	Axon guidance	8.45E-03	19
32	B cell receptor signaling pathway	9.21E-03	13
33	Lysine degradation	1.07E-02	10
34	Ubiquitin mediated proteolysis	1.28E-02	21
35	Synthesis and degradation of ketone bodies	1.31E-02	3
36	Bacterial invasion of epithelial cells	1.31E-02	12
37	Salmonella infection	1.31E-02	14
38	African trypanosomiasis	1.31E-02	7
39	Wnt signaling pathway	1.31E-02	23
40	MAPK signaling pathway	1.31E-02	35

41	HTLV-I infection	1.31E-02	37
42	Herpes simplex infection	1.50E-02	30
43	Hepatitis C	1.73E-02	19
44	Mismatch repair	2.11E-02	4
45	Acute myeloid leukemia	2.16E-02	9
46	SNARE interactions in vesicular transport	2.49E-02	7
47	Butanoate metabolism	2.69E-02	6
48	Fc gamma R-mediated phagocytosis	2.69E-02	14
49	HIF-1 signaling pathway	3.73E-02	17
50	Pathogenic Escherichia coli infection	4.38E-02	9

Table S5 Characteristics of diagnostic clinical trials included in the meta-analysis

Author (year)	Ethnicity	Country	Sample size		Sample source	Diagnostic tests	
			Case	Control		Sens	Spec
Wang (2009)	Caucasian	US	28	19	Blood	46.0	89.0
Sadakari (2010)	Asian	Japan	12	3	Digestive juice	91.7	100.0
Ryu (2011)	Asian	Korea	24	16	Digestive juice	76.0	80.0
Liu (2011)a	Asian	China	45	45	Blood	66.7	71.1
Liu (2011)b	Asian	China	45	30	Blood	77.8	66.7
Que (2013)	Asian	China	22	27	Blood	95.5	81.5
Abue (2014)a	Asian	Japan	32	30	Blood	75.0	73.0
Abue (2014)b	Asian	Japan	32	42	Blood	80.0	63.0
Humeau (2015)	Caucasian	France	7	4	Saliva	71.4	100.0

a. Patients with non-tumor benign disease as control; b. Healthy volunteer as control.
